# Mesoporous carbon spheres with programmable interiors as efficient nanoreactors for H_2_O_2_ electrosynthesis

**DOI:** 10.1038/s41467-024-45243-w

**Published:** 2024-02-01

**Authors:** Qiang Tian, Lingyan Jing, Hongnan Du, Yunchao Yin, Xiaolei Cheng, Jiaxin Xu, Junyu Chen, Zhuoxin Liu, Jiayu Wan, Jian Liu, Jinlong Yang

**Affiliations:** 1 Shenzhen Key Laboratory of Energy Electrocatalytic Materials, Guangdong Research Center for Interfacial Engineering of Functional Materials, College of Materials Science and Engineering, Shenzhen University, Shenzhen, China; 2 College of Physics and Optoelectronic Engineering, Shenzhen University, Shenzhen, China; 3 College of Chemistry and Environmental Engineering, Shenzhen University, Shenzhen, China; 4 State Key Laboratory of Catalysis, Dalian Institute of Chemical Physics, Chinese Academy of Sciences, Dalian, China; 5 Global Institute of Future Technology, Shanghai Jiaotong University, Shanghai, China

**Keywords:** Electrocatalysis, Structural properties

## Abstract

The nanoreactor holds great promise as it emulates the natural processes of living organisms to facilitate chemical reactions, offering immense potential in catalytic energy conversion owing to its unique structural functionality. Here, we propose the utilization of precisely engineered carbon spheres as building blocks, integrating micromechanics and controllable synthesis to explore their catalytic functionalities in two-electron oxygen reduction reactions. After conducting rigorous experiments and simulations, we present compelling evidence for the enhanced mass transfer and microenvironment modulation effects offered by these mesoporous hollow carbon spheres, particularly when possessing a suitably sized hollow architecture. Impressively, the pivotal achievement lies in the successful screening of a potent, selective, and durable two-electron oxygen reduction reaction catalyst for the direct synthesis of medical-grade hydrogen peroxide disinfectant. Serving as an exemplary demonstration of nanoreactor engineering in catalyst screening, this work highlights the immense potential of various well-designed carbon-based nanoreactors in extensive applications.

## Abstract (web summary)

Nanoreactors, with biomimetic features and distinct catalytic functions, show promise in catalytic energy conversion. Here the authors propose the utilization of precisely engineered mesoporous carbon spheres with tunable hollow sizes as nanoreactors, leveraging their catalytic functionalities for enhanced diffusion and microenvironment modulation effects to achieve efficient hydrogen peroxide electrosynthesis.

## Introduction

Natural reactors, such as cells and organelles, provide a micrometer-scale hollow space and a suitable internal microenvironment for biochemical processes^1^. Their well-organized interiors enable reactants to be oriented and induced in a regulated way to perform specific functions^2,3^. Inspired by these cellular structures, various artificial nanoreactors have been designed and synthesized, making them a popular catalytic material for extensive applications^4–6^. Among them, mesoporous hollow nanoreactors (MHNs) are a novel type of catalytic material. Their unique internal hollow space and mesoporous structure can accommodate complex catalytic processes^7–9^. In particular, the hollow space in MHNs provides a confined internal microenvironment, allowing for modulation of molecular catalytic behavior^10–12^. Additionally, the mesoporous channels could provide the opportunity to control molecular diffusion, adsorption, and surface reactions for tunable catalytic reaction pathway^13–15^.

The development of MHNs presents challenges in understanding reaction processes at micro/nano interface^16^. Nevertheless, MHNs possess the potential to modulate the reaction process, and its nanoscale structural parameters can be tailored to regulate mass transfer and microenvironment, thereby enabling the tailoring of catalytic reaction pathway^17–19^. Yet, the effect of structural parameters on overall catalytic kinetics is not fully understood due to the limitations in model material synthesis, which presents a significant challenge for improving catalytic activity, selectivity, and designing novel catalysts, as well as elucidating reaction mechanisms^20,21^.

Colloidal carbon spheres are preferred for constructing parameter-tunable nanoreactors due to their unique advantages, such as adjustable porous structures, controllable particle size, large surface area, and adjustable surface chemistry^22–24^. Specifically, innovative synthetic methodology tends to provide carbonaceous structures with a wide range of tunability for active sites, such as oxygen-containing functional groups^25,26^, defects^27,28^, and edge sites^29,30^. The properties exhibited by carbon spheres present novel opportunities for screening appropriate probe reactions, such as the electrochemical two-electron oxygen reduction reaction (2e^-^ ORR) for hydrogen peroxide (H_2_O_2_) production, and for exploring the operational mechanisms of nanoreactors^31,32^.

**Fig. 1:**
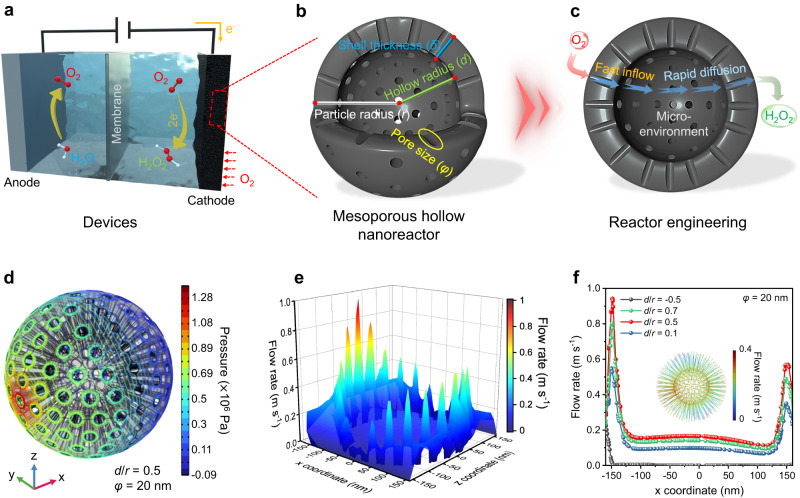
Optimization of diffusion-related geometric parameters in MHNs. **a**–**c** Schematic diagram of nanoreactor design based on carbon spheres for electrochemical 2e^-^ ORR to produce H_2_O_2_. **d** Spatial distribution of pressure contours for the mesoporous carbon sphere model (*d*/*r* = 0.5, *r* = 150 nm, *φ* = 20 nm). **e** 3D color mapping of the spatial flow velocity distribution on the cross-section. **f** Fluid velocity distribution across mesoporous hollow carbon sphere models (*φ* = 20 nm) with varying hollow percentage (*d*/*r* = −0.5, 0.1, 0.5, and 0.7). Inset in **f** shows flow linear distribution within the hollow mesoporous sphere model.

Understanding the operational mechanisms of nanoreactors may drive the on-site electrosynthesis of practical H_2_O_2_ solutions as an oxidant, disinfectant, and emerging energy fuel (Fig. 1a)^33–35^. In addition, sustainable H_2_O_2_ electro-production eliminates the multi-step, high-energy input, and environmental impacts associated with the traditional anthraquinone (AQ) industry^36^. Efficient electrosynthesis of H_2_O_2_ relies on electrocatalysts that facilitate the highly active 2e^-^ pathway^37^. Among the reported electrocatalysts, carbon-based materials have emerged as a promising alternative due to their abundance, low cost, and tunable catalytic properties^38^. To achieve high 2e^-^ ORR performance, the surface of carbon material must effectively activate O_2_ and maintain a suitable binding energy to *OOH intermediates^39,40^. Additionally, the carbon structure should possess a diffusion-friendly geometry to enable rapid separation of the generated H_2_O_2_ from the catalyst layer, preventing its further electro-reduction^41,42^. These challenges pose significant obstacles to the practical implementation of carbon materials for electrochemical 2e^-^ ORR. Nevertheless, by employing nano-engineering techniques to manipulate the structure of carbon sphere materials and harnessing the catalytic functions, including diffusion and microenvironmental modulation within nanoreactors, there is a promising opportunity to overcome these challenges and advance practical strategies for H_2_O_2_ electrosynthesis.

In this work, we proposed the utilization of meticulously controlled carbon spheres as the fundamental building material and employed electrochemical 2e^-^ ORR as a diffusion-related probe reaction to validate the catalytic functionality and superior performance of MHNs. The investigation began with finite element simulations (FES) to model the fluid behavior within the mesopores and hollow spaces of MHNs. Through precise tailoring of the internal structures of carbon spheres, a series of carbon-based MHNs was constructed as a proof of concept. The electrochemical properties and simulation results confirmed the enhanced diffusion and microenvironment-modulating effects of MHNs, particularly when designed with an appropriate hollow size. Leveraging these catalytic functionalities of the nanoreactor, the optimized MHCS_0.5_ catalysts exhibited impressive activity (−3.1 mA cm^−2^ at pH 13; −2.8 mA cm^−2^ at pH 7) and selectivity (>95% at pH 13; >85% at pH 7) in H_2_O_2_ electrosynthesis conducted by rotating ring-disk electrode method. Moreover, the successful production of medical-grade H_2_O_2_ disinfectant in a flow cell device highlighted the significant practical potential of nanoreactor engineering. Therefore, our work can serve as a paradigm for advancing the design and understanding of nanoreactor construction and its application in the practical electrochemical production of H_2_O_2_ solutions.

## Results

### Optimizing diffusion-related geometrical parameters of MHNs

We endeavored to employ engineered carbon spheres to construct MHNs for the purpose of achieving efficient H_2_O_2_ electrosynthesis. Such MHNs typically consist of a mesoporous shell layer and an internal hollow, which can be calibrated with several crucial geometric parameters including particle radius (*r*), pore size (*φ*), shell thickness (*δ*), and hollow radius(*d*) (Fig. 1b). These structural parameters may alter the fluid dynamics within MHNs, thereby regulating local diffusion and microenvironment, and consequently influencing the reaction process (Fig. 1c).

To investigate the correlation between the nanoreactor architecture and the fluid behavior inside, FES was conducted^43,44^. To ensure systematic construction of the MHNs and conserve computational resources for investigating the regularity of the electrolyte fluid, we standardized the particle size to 300 nm while maintaining the principle of unique variability for different *d*/*r* or *φ*. As shown in Supplementary Fig. 1a, b, a mesoporous hollow sphere model (*d*/*r* = 0.5, *r* = 150 nm, *φ* = 20 nm) was constructed in three-dimensional (3D) square space (320 nm × 320 nm × 320 nm). To simulate fluid flow in an agitated system, the inlet and outlet of the fluid field were set as the left and right sides, respectively (Supplementary Fig. 1c, d). Such a setup generates a decreasing pressure gradient from left to right that drives water to flow spontaneously through the constructed geometry (Supplementary Fig. 2)^13^. Interestingly, there is a noticeable pressure drop inside the mesopores (*φ* = 20 nm) relative to the surface of the non-porous sphere (Supplementary Figs. 3 and 4). The pressure difference across the mesopores drives water flow through the channels and into the sphere interior, greatly expanding the available catalytic surface by increasing the contact between the electrolyte and carbon surface (Fig. 1d and Supplementary Fig. 5). More interestingly, driven by this pressure difference, a significant fluid acceleration appears in the mesoporous channels (*φ* = 20 nm), implying an intensified diffusion in MHNs (Supplementary Figs. 6 and 7). The 3D mapping of fluid velocity in the central cross-section (y = 0) confirms the local diffusion intensification within mesoporous channels and indicates the potential for rapid delivery of O_2_ and exhaustion of generated H_2_O_2_ (Fig. 1e)^13^.

To evaluate the diffusion effect and identify the optimal solution, a range of pore size parameters (*φ* = 0, 2 nm, 5 nm, 10 nm, 20 nm, and 30 nm) were simulated, along with calculating the fluid velocity distribution at specific locations (−160, 0, 0 → 160, 0, 0) to facilitate comparison and analysis (Supplementary Fig. 6b). The highest flow rates were observed in both the inflow and outflow mesopore channels at a pore size of 20 nm, despite the slower flow rate in the mesopore-linked hollow space (Supplementary Fig. 8–10). Based on the above, we continue to maintain similar particle parameters but vary the hollow ratios for predicting fluid mass transfer. So, models with different hollow occupancy ratios (*d*/*r* = −0.5, 0.1, 0.5, and 0.7) are also simulated (Supplementary Figs. 9 and  11). The results indicate that a certain size of hollow space is crucial, with the maximum flow velocity in the mesoporous channels occurring at *d*/*r* = 0.5 (Fig. 1f). Considering that the active sites for the electrochemical ORR are dispersed on the carbonaceous surface of the mesoporous shells, the localized diffusion enhancement enables the swift inflow of O_2_ and the rapid transfer of the generated H_2_O_2_ into the bulk solution, thereby averting its accumulation on the catalyst surface and subsequent electro-reduction^45^. These findings indicate that the density of mesopores may influence the fluid behavior within the MHNs. For a single MHN, a small *d*/*r* ratio suggests a long mesoporous channel and high mesoporous density, resulting in continuous viscous resistance that affects the flow rate^13,46^. Conversely, a large *d*/*r* implies a short mesoporous channel and a larger hollow structure with lower mesoporous density, enabling rapid fluid entry into the hollow’s flow rate buffer zone, though limiting fluid acceleration. Additionally, the modified MHN model, constructed with reduced mesopore density, demonstrates the electrolyte’s ability to maintain a high flow rate over a long distance (Supplementary Fig. 12). However, the decrease in mesoporous channels would cause most of the electrolyte to flow from the MHN’s surface rather than through the mesopores, potentially reducing the utilization of the internal active sites. The fluid buffering effects in the hollow region may establish a potentially stable internal microenvironment. To summarize, micromechanical dynamics simulations were employed to identify diffusion-favorable structural parameters (*d*/*r* = 0.5, *φ* = 20 nm) and guide the design of MHNs.

### Refinement and characterization of carbon-based MHNs

**Fig. 2:**
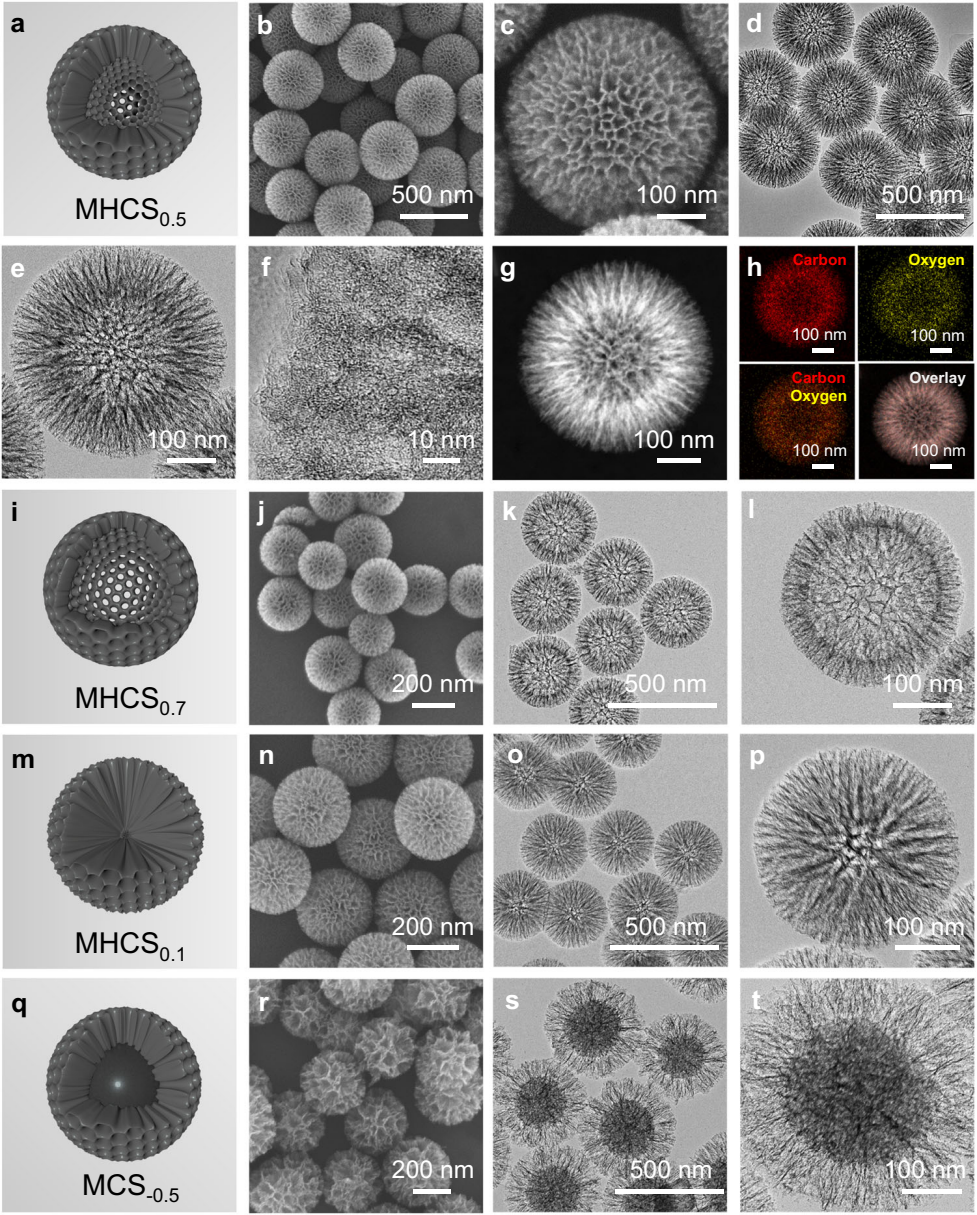
Electron microscopy images of synthesized carbon-based MHNs. **a** Structural model, **b,** **c** SEM images, **d,** **e** TEM images, **f** HRTEM image, **g** HAADF-STEM image, and the corresponding **h** elemental mapping images of MHCS_0.5_. **i** Structural model, **j**, SEM image, and **k,** **l** TEM images of MHCS_0.7_. **m** Structural model, **n** SEM image, and **o,** **p** TEM images of MHCS_0.1_. **q** Structural model, **r** SEM image, and **s**, **t** TEM images of MCS_−0.5_.

Inspired by the simulation results above, a sequential organic-inorganic hybridization co-assembly approach was developed to fabricate mesoporous hollow carbon sphere-based nanoreactors. As shown in Supplementary Fig. 13, this approach begins with the chronological modular assembly of organic phenolic resin and inorganic silica (SiO_2_)^47,48^. In detail, resorcinol and formaldehyde are polymerized under the catalyst of ammonia to form phenolic resins, while tetrapropoxysilane (TPOS) added simultaneously underwent hydrolysis to form SiO_2_. And then, these two components spontaneously co-assemble into the hybrid resin/SiO_2_ spheres (Supplementary Fig. 14a, b). After further pyrolysis of resin/silica spheres in an inert atmosphere, the resin zone is converted into carbon components, resulting in the formation of C/SiO_2_ spheres (Supplementary Fig. 14c, d). The carbonaceous material in the C/SiO_2_ spheres was selectively eliminated by calcination under an air atmosphere, yielding mesoporous silica spheres (MSiO_2_S) with a solid core, providing compelling evidence that TPOS was initially hydrolyzed to form a SiO_2_ core, which was subsequently surrounded by the hybridization of resin and SiO_2_ (Supplementary Fig. 15). Thus, the selective removal of SiO_2_ component from the C/SiO_2_ spherical precursor led to the formation of mesoporous hollow carbon spheres (MHCS_x_, x = *d*/*r*) by generating mesopores and hollow in the areas previously occupied by the SiO_2_ phase^49^. The morphology of the MHCS_x_ was analyzed by SEM and TEM, revealing an average particle radius of 210 nm and a hollow radius of 105 nm, resulting in the designation of MHCS_0.5_ (Fig. 2a–e). Further, high-resolution transmission electron microscopy (HRTEM) indicated the permeable mesoporous channels on the external shell (Fig. 2f). Besides, some mesoporous channels running through the shell layer were clearly observed in both the SEM images of the crushed MHCS_0.5_ and the locally enlarged TEM images of MHCS_0.5_, providing a path for the electrolyte fluid to pass through the internal hollow space (Supplementary Fig. 16). Additionally, the high-angle annular dark-field scanning transmission electron microscopy (HAADF-STEM) image confirms the hollow mesoporous architecture of MHCS_0.5_, as well as the even distribution of carbon and oxygen elements (Fig. 2g, h). To confirm the flow of electrolyte through the internal hollow space of MHCS_0.5_, we conducted an initial one-hour ORR using MHCS_0.5_ in 0.5 M KCl electrolyte. Following the reaction, the MHCS_0.5_ samples were dried without washing for direct TEM observation, revealing the presence of residual KCl components filling the interiors of MHCS_0.5_ (Supplementary Fig. 17). These findings are further validated by the corresponding elemental linear scans, confirming the presence of mesoporous channels running through the shell layers of MHCS_0.5_ and the flow of electrolyte fluids through these channels, enabling movement of electrolyte through the hollow interior (Supplementary Fig. 18).

In the sequential organic-inorganic hybridization co-assembly strategy, the timing of resin and SiO_2_ assembly can be flexibly manipulated to significantly adjust the size of the hollow structure of MHCS. We attempted to manipulate the time nodes on formation of resin and SiO_2_ to synthesize mesoporous spheres with varying hollow sizes. To achieve this, we added resin monomers (resorcinol and formaldehyde) first, followed by the addition of TPOS after a specific time interval (*t* min). For the synthesis of MHCS_0.5_, the value of *t* was controlled to be 0, implying that the resin monomers and the TPOS required for hydrolysis to form SiO_2_ were added simultaneously. If TPOS is added 20 min earlier (*t* = −20), the hydrolysis process of TPOS is advanced, leading to the formation of larger SiO_2_ solid core before it is hybridized with the resin. After carbonization and SiO_2_ removal, MHCS_0.7_ with larger hollow size than MHCS_0.5_ was synthesized (Fig. 2i–l). By delaying the addition of silane by 20 min (*t* = 20), the hybridization co-assembly of SiO_2_ with resin can be advanced correspondingly, resulting in the formation MHCS_0.1_ with an ultra-small hollow (Fig. 2m–p). Further, by adding the resin monomer for 80 min and then hydrolyzing TPOS (*t* = 80), a resin solid core is first polymerized and then surrounded by a hybridization of SiO_2_ and resin, resulting in the formation of mesoporous carbon spheres (named MCS_−0.5_) with solid carbon cores and mesoporous carbon shells (Fig. 2q–t). The geometrical parameters of the synthesized series of MHCS demonstrated a clear positive correlation between the ratio of hollow size to particle size (*d*/*r*) and the *t* value, providing evidence for the controlled synthesis of hollow mesoporous nanoreactors under time order manipulations (Supplementary Table. 1 and Supplementary Fig. 19).

Some comparative experiments were also conducted to confirm the role of SiO_2_, derived from the hydrolysis of TPOS, in the formation of hollow mesoporous carbon spheres. Without the addition of TPOS, only the polymerization of phenolic resin would occur, resulting in the formation of non-mesoporous resin spheres (Supplementary Fig. 20). Pyrolysis of these resin spheres would then produce microporous carbon spheres (CS, Supplementary Fig. 21). Additionally, by burning off the resin components of resin/SiO_2_ spheres assembled at various time intervals (*t* = −20, 20 and 80) in the air atmosphere, various mesoporous spherical SiO_2_ structures with the topologies opposite to that of the corresponding MHCS_x_ (MHCS_0.7_, MHCS_0.1_ and MCS_−0.5_) were obtained (Supplementary Fig. 22–24). These findings substantiate the controlled manipulation of hierarchical heterogeneous assembly of SiO_2_ and resin in a specific temporal order.

**Fig. 3:**
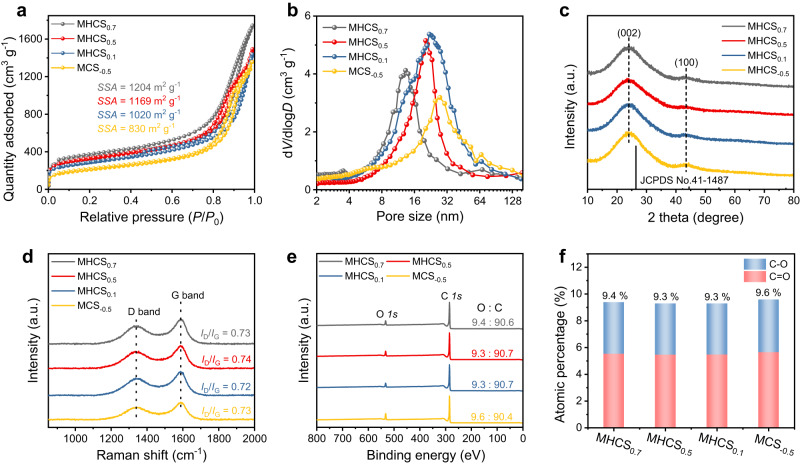
Characterization of as-obtained MHNs. **a** Nitrogen adsorption-desorption isotherms, **b** pore size distribution curves, **c** XRD patterns, **d** Raman spectra, **e** XPS survey spectra, and the corresponding **f** C-O/C = O species content of MHCS_x_ samples.

We analyzed the porous structure and physicochemical characteristics of five samples: MCS_−0.5_, MHCS_0.1_, MHCS_0.5_, MHCS_0.7_, and CS. The nitrogen adsorption and desorption isotherms revealed high Brunauer-Emmett-Teller (BET) specific surface areas (SSA) of 830, 1020, 1169, and 1204 m^2^ g^−1^ for MCS_−0.5_, MHCS_0.1_, MHCS_0.5_, and MHCS_0.7_, respectively (Fig. 3a). The Barrett-Joyner-Halenda method revealed the mesopore size distribution of MHCS_x_ samples to be in the range of 15–25 nm (Fig. 3b). Compared to MHCS_x_, CS has a lower specific surface area of only 552 m^2^ g^−1^ and almost no mesopore distribution (Supplementary Fig. 25). XRD patterns of MHCS_x_ and CS showed broad peaks at 23.0° and 43.6°, corresponding to the (002) and (100) planes of the disordered carbonaceous structure, respectively (Fig. 3c and Supplementary Fig. 26a)^50^. The Raman spectra showed similar intensity ratios of D and G bands and a G linewidth of 100 cm^−1^ for all five samples, indicating typical characteristics of amorphous carbon with comparable defects (Fig. 3d and Supplementary Fig. 26b)^51^. XPS analysis revealed an oxygen content of around 9.5 at% for MHCS_x_ (Fig. 3e). The C *1* *s* spectrum shows three peaks at 284.6, 286.0, and 288.7 eV, corresponding to C − C, C − O, and O − C = O, respectively, while the O *1* *s* spectrum exhibits two peaks assigned to C = O (531.6 eV) and C − O (533.0 eV) (Supplementary Fig. 27 and Supplementary Fig. 28)^43^. Fig. 3f and Supplementary Table. 2 shows that the distribution of oxygen-containing functional groups, which are considered as active sites for electrochemical 2e^-^ ORR^25,26,52,53^, is similar among the different MHCS_x_ samples. Besides, CS had a similar chemical composition to MHCS_x_ (Supplementary Fig. 29). In addition, defective carbon or some carbon edges, have been proposed as potential active sites for 2e^-^ ORR in some previous studies^30,54^. To examine the defective carbon, we utilized electron paramagnetic resonance (EPR) and observed nearly identical signal intensities on MHCS_x_, suggesting that MHCS_x_ exhibits a consistent level of defectivity for electrochemical 2e^-^ ORR (Supplementary Fig. 30). These similar properties observed in MHCS_x_ may be attributed to their shared origin as resorcinol-formaldehyde resin-derived carbon and undergoing the same carbonization process. Therefore, these as-obtain carbon spheres have similar chemical properties and active sites, with differences mainly in hollow size and thickness of mesoporous shells, making them ideal models for exploring the efficacy of MHNs for catalytic reactions.

### Electrocatalytic performance

**Fig. 4:**
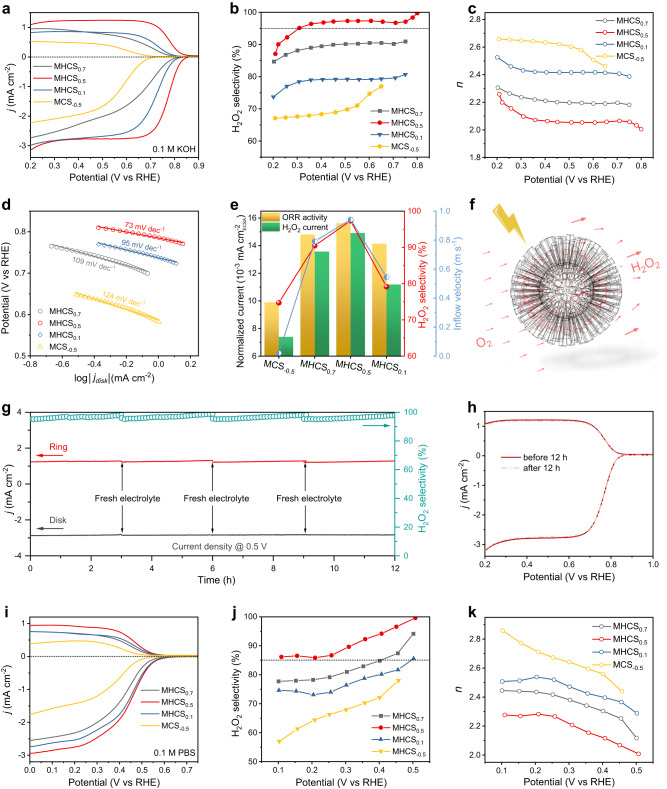
Electrochemical performance of MHNs. **a** LSV curves of RRDE measurements at 1600 rpm in 0.1 M KOH, **b** the corresponding H_2_O_2_ selectivity, and **c** electron transfer number at various applied potentials. **d** Tafel plots derived from LSV curves in 0.1 M KOH. **e** Normalized electrochemical activity and H_2_O_2_ partial current on MHCS_x_ (@ 0.6 V vs RHE), along with the corresponding H_2_O_2_ selectivity and electrolyte inflow velocity. **f** Schematic diagram depicting the flow of fluid/oxygen and the oxygen reduction process on the MHCS_0.5_ model. **g** Stability measurements of MHCS_0.5_ at a fixed disk potential of 0.5 V vs RHE in 0.1 M KOH. **h** LSV curves of MHCS_0.5_ before and after 12 h stability test. **i** LSV curves of RRDE measurements at 1600 rpm in 0.1 M PBS, **j** the corresponding H_2_O_2_ selectivity, and **k** electron transfer number at various applied potentials.

As a proof of concept, the electrochemical 2e^-^ ORR was used as a probe reaction to evaluate catalytic process intensification due to fluid transport in this series of carbon-sphere based MHNs. The electrochemical performance was evaluated using a three-electrode rotating ring-disk electrode (RRDE) technique at 1600 rpm in an O_2_-saturated electrolyte, with the Pt ring electrode set at 1.2 V vs reversible hydrogen electrode (RHE) to detect the produced H_2_O_2_ on the disk. Remarkably, the MHCS_0.5_ achieves a large diffusion-limiting disk current density (−3.1 mA cm^−2^ at 0.20 V vs RHE) in 0.1 M KOH (pH = 13), approaching the theoretical limit for 2e^−^ ORR (Fig. 4a)^41,55^. Besides, the MHCS_0.5_ delivers a high onset potential of 0.85 V vs RHE, larger than the thermodynamic equilibrium 2e^-^ ORR potential (0.75 V)^52,56,57^. The high ORR onset potential may stem from its appropriate porous structure and geometric framework, which enhance contact with electrolyte fluids to improve the utilization of carbon surface active sites in the electrolyte environment^14,19,58^. Correspondingly, the electrochemical activity of non-mesoporous CS was found to be poor, as indicated by a very low disk current densities at diffusion-limiting conditions, suggesting the insufficient the exposure of active sites in the micropores (Supplementary Fig. 31). Figure 4b, c shows the calculated H_2_O_2_ selectivity and the corresponding electron transfer number (*n*) for MHCS_x_ samples. The MHCS_0.5_ catalyst delivers the high H_2_O_2_ selectivity (> 95%) and a *n* value around 2.05 across a wide potential window (~0.5 V, from 0.3 V to 0.8 V vs RHE), which are superior to those of most of the previously reported catalysts (Supplementary Table. 3). Decreases in H_2_O_2_ selectivity were observed in the order of MHCS_0.5_, MHCS_0.7_, MHCS_0.1_, and MCS_−0.5_, showing a positive relationship with the simulated flow rate in the mesopore channels of the corresponding MHNs models.

To comprehend the distinctions in the electrochemical properties of MHCS_x_, it is imperative to uncover the intrinsic active sites involved in electrocatalytic ORR. We reduced the MHCS_0.5_ sample in a mixed H_2_/N_2_ atmosphere to eliminate the oxygen component, resulting in R-MHCS_0.5_ with an oxygen content as low as 1.76 at% (Supplementary Fig. 32a). Consequently, the ORR activity and H_2_O_2_ selectivity of R-MHCS_0.5_ were significantly reduced (Supplementary Fig. 32b), indicating that the oxygen species on the carbon matrix serve as the active source of 2e^-^ ORR, consistent with some previous reports^52,59^. Given the comparable oxygen species on MHCS_x_, the notable discrepancy in H_2_O_2_ selectivity is attributed to the fluid behavior of electrolytes within different architectures of MHNs. Fig. 4d displays the Tafel slopes of MHCS_x_, with MHCS_0.5_ exhibiting a low value of 73 mV dec^−1^, indicating its fast reaction kinetics. Tafel plots reflect various rate-determining steps (RDS), and a progressive decrease in Tafel slopes could indicate a shift in the RDS from the first electron-transfer step (O_2_ → *OOH, ~120 mV dec^−1^) to the last H_2_O_2_ desorption step (*OOH → H_2_O_2_, ~ 70 mV dec^−1^)^60,61^. The ORR pathway was studied from the perspective of the H_2_O_2_ reduction reaction (H_2_O_2_RR) activity in N_2_-saturated 0.1 M KOH containing 10 mM H_2_O_2_. Among MHCS_x_, MHCS_0.5_ exhibits apparent inertness to H_2_O_2_RR, with negligible currents, confirming its highly selective 2e^-^ pathway (Supplementary Fig. 33). In contrast, we observed an increasing trend in H_2_O_2_RR current from MHCS_0.7_, MHCS_0.1_ to MCS_−0.5_, suggesting an improved H_2_O_2_RR capability. Considering the simulated fluid velocity on MHCS_x_, we deduce that the low flow rates would advance the indirect 4e^-^ pathway to reduce the H_2_O_2_ selectivity^61,62^.

These carbon sphere-based electrocatalyst were further evaluated for their accessible active surface area (ECSA) using a double-layer capacitance (*C*_dl_) method. Specifically, the *C*_dl_ values for MHCS_0.5_, MHCS_0.1_, MHCS_0.7_, and MCS_−0.5_ were calculated to be 8.04, 7.20, 4.36, and 3.17, respectively (Supplementary Fig. 34, 35). Interestingly, the *C*_dl_ values of MHCS_x_ do not align with their total BET surface area, but instead, exhibit a positive correlation with the external surface area excluding the microporous region (Supplementary Fig. 35b)^63^. Considering the particle morphology of MHCS_x_, it can be hypothesized that the internal surface exposure, facilitated by the mesoporous channels, enhances the interaction between the electrolyte and active sites, thereby promoting heightened electrocatalytic activity^64,65^. Prominently, the high *C*_dl_ value on MHCS_0.5_ signifies its structurally favorable diffusion, suggesting a swift supply of electrolyte ions and effective utilization of active sites. Besides, the non-mesoporous CS exhibits a low *C*_dl_ of 2.38 mF cm^−2^, further illustrating the restricted accessibility of micropores by electrolytes (Supplementary Fig. 36)^54^. Furthermore, we utilized ECSA to standardize the electrochemical activity and the H_2_O_2_ partial currents of MHCS_x_ at diffusion-related potentials (0.6 V vs RHE), as illustrated in Fig. 4e. Interestingly, the associated normalized currents and 2e^-^ selectivity exhibit a positive correlation with the simulated electrolyte inflow velocity, providing additional evidence for the connection between the diffusion effect and the electrochemical performance on these MHNs. These characteristics demonstrate the influence of the hollow’s structural parameters on diffusion and their reflection on electrocatalytic performance (Fig. 4f).

To assess the catalytic stability, we conducted chronoamperometric tests for MHCS_0.5_ using the RRDE technique at a constant disk potential of 0.5 V vs RHE. During the continuous testing, the accumulated H_2_O_2_ might lead to a slight rise in the ring current and subsequently in selectivity. To maintain a consistent ring current, we replaced the electrolyte every 3 h and performed electrochemical cleaning of the Pt ring to remove any effects of the accumulated H_2_O_2_ during continuous operation. The results showed that the MHCS_0.5_ catalyst maintained stable disk/ring currents and achieved over 95% selectivity throughout the 12-hour continuous test (Fig. 4g). Further, we conducted an accelerated durability test (ADT) on MHCS_0.5_ by sweeping the potential between 0.2 and 1.0 V vs RHE for 12 h. The similar linear sweep voltammetry curves obtained before and after ADT confirm its high stability (Fig. 4h). After long-term electrolysis, MHCS_0.5_ still maintains its hollow mesoporous structure, and its chemical composition remains consistent with its pre-reaction state, ensuring its stability and reusability (Supplementary Figs. 37 and 38). Of particular interest, MHCS_0.5_ demonstrates exceptional 2e^-^ ORR performance in neutral electrolyte conditions (pH 7), with a diffusion-limited disk current of −2.8 mA cm^−2^ at 0.2 V vs RHE, an onset potential of 0.6 V vs RHE, and an H_2_O_2_ selectivity exceeding 85%, surpassing vast majority of carbon-based catalysts reported in the literature (Fig. 4i–k and Supplementary Table. 4). The trends observed in the ORR activity and selectivity of MHCS_x_ under neutral conditions were consistent with those observed under alkaline conditions. Interestingly, the polarization curves exhibit two distinct sets of slopes at different potentials, suggesting variations in the ORR mechanism. Consistently, the H_2_O_2_RR test conducted on MHCS_0.5_ revealed a low H_2_O_2_RR current density, indicating its overall inertness to H_2_O_2_RR in a neutral electrolyte, with a slight increase observed at more negative potentials (Supplementary Fig. 39). This trend in the H_2_O_2_RR current aligns with that of the ORR, highlighting the influence of the indirect 4e^-^ process at relatively high voltages on the 2e^-^ selectivity^61^.

### Actual H_2_O_2_ electrosynthesis properties

The practical application of MHCS_0.5_ catalysts was evaluated using a H-type cell setup in 0.1 M KOH with continuous O_2_ bubbling (Supplementary Fig. 40a). The produced H_2_O_2_ was quantified by the Ce^4+^ titration method with the ultraviolet-visible (UV–vis) spectrophotometric technique. During a long-term stability test at a fixed voltage of 0.5 V vs RHE, the current remains stable at about 5.4 mA cm^−2^ and the concentration of accumulated H_2_O_2_ increases continuously, resulting in 40.29 mmol L^−1^ within 12 h (Supplementary Fig. 40b). During the long-term continuous test, the fluctuations of current and Faraday efficiency (FE) (> 97%) are slim, indicating the reliable practicality of the bulk MHCS_0.5_ electrocatalyst.

**Fig. 5:**
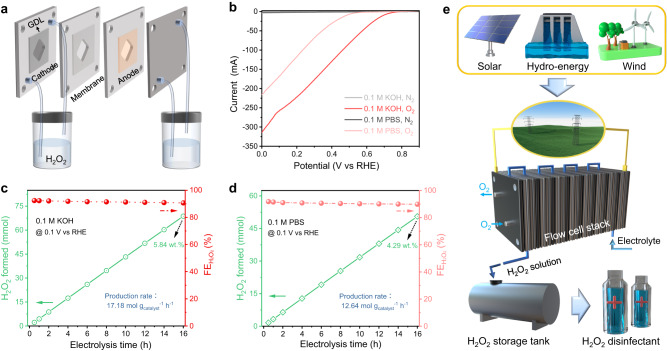
Actual H_2_O_2_ electro-production based on MHNs. **a** Schematic diagram of the flow cell for H_2_O_2_ electrosynthesis. **b** Polarization curves of MHCS_0.5_ loaded CP electrode in flow cell. **c** Electrolysis time-dependent H_2_O_2_ production and faradaic efficiency of MHCS_0.5_ under continuous O_2_ purging in 0.1 M KOH. **d** Electrolysis time-dependent H_2_O_2_ production and faradaic efficiency of MHCS_0.5_ under continuous O_2_ purging in 0.1 M PBS. **e** Envisioned green electricity-driven large-scale electrochemical production of H_2_O_2_ disinfectant.

To address the limitations of peroxide generation in a static H-cell configuration, characterized by inefficient mass transport due to low O_2_ diffusion efficiency and H_2_O_2_ desorption rate, leading to suboptimal apparent productivity,^66,67^ we further evaluated the actual H_2_O_2_ production from MHCS_0.5_ using a flow cell setup (Fig. 5a and Supplementary Fig. 41). In the measurements, a MHCS_0.5_ loaded carbon paper (CP) electrode, including a gas diffusion layer (GDL), was used as the cathode, and a commercial IrO_2_ served as the anode in the flow cell. As depicted in Fig. 5b, MHCS_0.5_ exhibited a sharp increase in current density with rising voltage, reaching ~254 mA in 0.1 M KOH and ~188 mA in 0.1 M phosphate buffered solution (PBS) at an uncompensated voltage of 0.1 V vs RHE. Moreover, the current of MHCS_0.5_ showed a consistent and steady profile during continuous electrolysis at an applied voltage of 0.1 V vs RHE (Supplementary Fig. 42). Notably, the production yield of H_2_O_2_ exhibited a steady increase over time, with a 16-hour cumulative yield of H_2_O_2_ reaching 68.7 mmol in 0.1 M KOH and 50.6 mmol in 0.1 M PBS, corresponding to H_2_O_2_ production rates of 17.18 and 12.64 mol g_catalyst_^−1^ h^−1^ (Fig. 5c, d) and outperforming most recently reported electrocatalysts (Supplementary Table. 5). Additionally, the Faraday efficiency of H_2_O_2_ electrosynthesis on MHCS_0.5_ remained consistently around 90% throughout a long-term test. After 16 h of electrosynthesis in the flow cell, the cumulative concentration of H_2_O_2_ reached 5.84 wt.% in an alkaline environment and 4.29 wt.% in a neutral medium, satisfying the concentration requirements for the medical-grade disinfectant (~ 3 wt.%)^68^. Notably, the electroproduction of H_2_O_2_ solutions in neutral electrolytes is particularly appealing due to its environmental friendliness, minimal corrosiveness, and potential for reduced electrolytic cell costs^69^. Additionally, neutral H_2_O_2_ solutions provide a sustainable and versatile option for practical applications, including the direct use of synthesized H_2_O_2_ in biochemical systems^70,71^. The robust performance and excellent selectivity for 2e^-^ reduction demonstrated by MHCS_0.5_ in the flow cell setup validate its potential for practical applications in electrochemical H_2_O_2_ production, particularly for the electro-production of neutral H_2_O_2_ solutions^37^. In contrast, MCS_−0.5_ exhibited restricted electrochemical activity, with currents of 51 mA at 0.1 M KOH and 25 mA at 0.1 M PBS at an applied potential of 0.1 V vs RHE (Supplementary Fig. 43a). Additionally, the FE of MCS_−0.5_ for H_2_O_2_ electro-production is approximately 60%, significantly lower than that of the MHCS_0.5_ electrode (Supplementary Fig. 43b, c). This noticeable distinction in electrochemical performance further underscores the effectiveness of the engineered carbon sphere-based MHNs.

Based on the remarkable activity, high H_2_O_2_ selectivity, and reliable stability of MHCS_0.5_, coupled with the global goal of carbon neutrality, we envision a future where large-scale production of H_2_O_2_ disinfectants is achieved using sustainable energy sources^67,72^. As depicted in Fig. 5e, surplus green electricity generated from solar, hydro, and wind energy sources is utilized as the energy supply for decentralized H_2_O_2_ production in an electrolytic cell stack. Significantly, the medical-grade H_2_O_2_ solution produced under neutral conditions can be elegantly stored or directly employed for disinfection and sterilization purposes. This green electricity-driven H_2_O_2_ disinfectant production represents a sustainable approach that enables on-site production and utilization of H_2_O_2_, circumventing the pollution, high energy consumption, and explosion hazards associated with the traditional AQ industry. In the pursuit of this goal, the nanoreactor engineered carbon spheres will serve as pivotal catalysts in realizing this vision.

### Microenvironmental modulation effect of nanoreactors

**Fig. 6:**
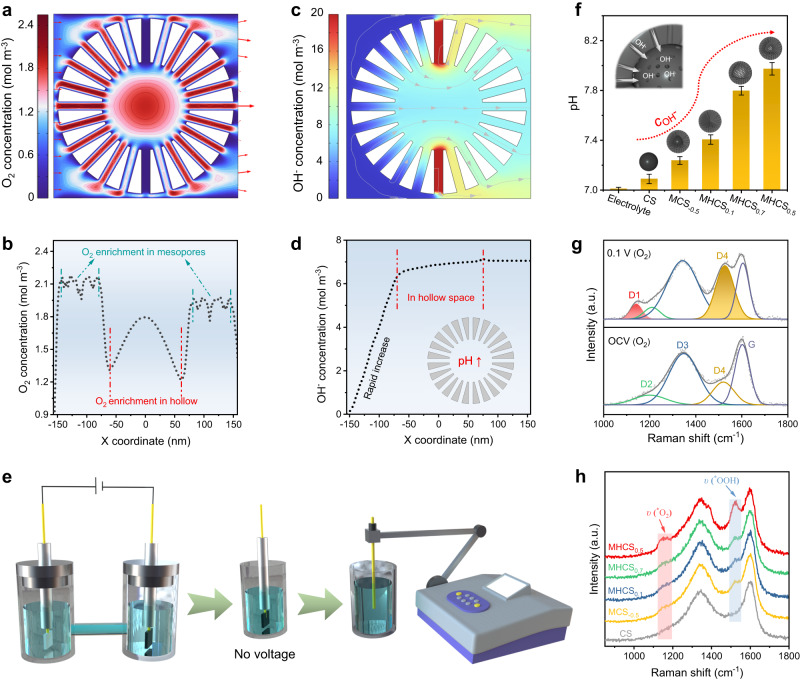
Microenvironmental modulation in MHNs. **a** Spatial distribution of O_2_ concentration in the mesoporous carbon sphere model (*d*/*r* = 0.5, *r* = 300 nm, *φ* = 20 nm), and **b** the corresponding O_2_ concentration distribution curve of mesoporous carbon sphere model on y = 0. **c** Spatial distribution of OH^-^ concentration of the mesoporous carbon sphere model (*d*/*r* = 0.5, *r* = 300 nm, *φ* = 20 nm), and **d** the corresponding OH^-^ concentration distribution curve of mesoporous carbon sphere model on y = 0. **e** Schematic diagram of the process for testing the OH^-^ concentration on the electrode surface. **f** Comparison of OH^-^ concentrations on the surface of MHCS_x_. **g** In-situ Raman spectra of MHCS_0.5_ electrode at open circuit voltage and applied potential (0.1 V vs RHE) in 0.1 M PBS. **h** Comparison of in-situ Raman spectra on MHCS_x_ electrode at applied potential (0.1 V vs RHE).

To gain a deeper understanding of the exceptional 2e^-^ ORR performance of MHCS_0.5_, particularly in neutral conditions, we conducted extensive simulations to elucidate the catalytic mechanism of the hollow mesoporous nanoreactor. To provide a visual representation of the interior within the nanoreactor, a two-dimensional (2D) model of a hollow mesoporous sphere (*d*/*r* = 0.5, *r* = 150 nm, *φ* = 20 nm) was constructed^49,73^. As shown in Supplementary Fig. 44, the mesoporous hollow sphere is modeled as having all surfaces as catalytically active sites and placed within a square simulation domain (320 nm × 320 nm), with the inflow and outflow boundaries for the electrolyte set at the left and right sides. Consistent with the results of the previous 3D simulations, the O_2_-containing electrolyte fluid is driven by the pressure gradient to flow through the mesopore-hollow-mesopore of the sphere and ultimately exits the simulation domain (Supplementary Fig. 45). We obtained the velocity distribution at y = 0 and observed a significant acceleration of the fluid flow in the mesopore region, while the flow velocity within the hollow region was relatively low (Supplementary Fig. 46). High flow rates can increase the entrainment of O_2_ per unit time, as evidenced by the spatial concentration distribution of O_2_ being almost proportional to the flow rate distribution, leading to the enrichment of O_2_ in pore channels (Fig. 6a, b). From a reaction kinetics perspective, the local enrichment of O_2_ in the mesoporous channels can promote the activation of O_2_ (O_2_ + * → *O_2_) and stimulate the formation of *OOH as well as the consumption of protons to generate OH^-^ (*O_2_^-^ + H_2_O → *OOH + OH^-^)^67,74,75^. The accumulation of OH^-^ generated, which serves as a flow buffer, causes a local pH elevation within the MHNs, as confirmed by the finite element simulation results (Fig. 6c, d). After the electrolysis in neutral media (0.1 M PBS, pH = 7), phenolphthalein indicator was quickly dropped onto the MHCS_0.5_ electrode and inserted into a bottle containing neutral electrolyte. The appearance of a slight purplish red color on the electrolytic surface was observed, providing evidence for the presence of a localized pseudo-alkaline microenvironment (Supplementary Fig. 47). More directly, a significant in-situ color change was observed on MHCS_0.5_ electrode surface during a 2e^-^ ORR in neutral electrolyte (0.1 M K_2_SO_4_) containing phenolphthalein (Supplementary Fig. 48). This color change and the electrocatalytic process proceeded almost simultaneously, providing evidence of a local pH increase near the catalyst layer (Supplementary Movie 1). Next, we conducted the following experiments to verify the MHNs’ ability to accumulate OH^-^. Initially, as shown in Fig. 6e, the ORR process was performed for 10 min in a neutral electrolyte using a carbon sphere catalyst at 0.4 V vs. RHE, with the corresponding current density recorded in Supplementary Fig. 49a. Subsequently, all electrodes were extracted from the cell, and the working electrode was rinsed by immersion in 5 mL of an aqueous solution. This process was repeated 10 times, and the pH of the aqueous solution was measured. Fig. 6f illustrates that the MHCS_0.5_ electrode surfaces exhibited higher OH^-^ concentrations compared to the other samples. This observation is associated with the high current density generated by the MHCS_0.5_ electrodes, leading to increased production of OH^-^ by-products. Additionally, it was observed that there is no linear relationship between the current densities and measured OH^-^ concentrations for each carbon sphere electrode (Supplementary Fig. 49b, c). Rather, the electrode with the hollow-structured carbon sphere exhibited a significantly higher OH^-^ concentration. Therefore, we attribute the localized OH^-^ accumulation on the MHCS_0.5_ electrode to the combined effects of its high electrochemical activity and favorable hollow geometric configuration. These results provide experimental evidence that supports the simulation results discussed above.

To investigate the effect of O_2_ enrichment and local pH elevation on the 2e^-^ ORR within the carbon sphere-based MHNs, we utilized in-situ Raman spectroscopy to probe the catalyst-adsorbate interactions (*O_2_, *OOH). In an oxygen atmosphere, the Raman spectra of a MHCS_0.5_ electrode at the open-circuit voltage (OCV) and an applied potential of 0.1 V vs RHE are shown in Fig. 6g. The D band was fitted by the characteristic modes of D1, D2, D3, and D4 at ~1130, 1200, 1340, and 1500 cm^−1^, respectively^74^. Compared with the OCV, the D peak widened and a D1 peak emerged, which reflects the adsorption of O_2_ (*O_2_), and a corresponding sharper D4 peak indicates a greater formation of *OOH intermediate^52,76,77^. This phenomenon can be inferred as follows: the enrichment of O_2_ and localized pH elevation in the MHNs accelerate the reaction rate, facilitating the activation of O_2_, stabilizing *O_2_ intermediates, and inhibiting the protonation process of *OOH intermediates (*OOH + H^+^ + e^-^ → *O + H_2_O) associated with the 4e^-^ pathway, thereby promoting H_2_O_2_ generation^74,78^. As illustrated in Fig. 6h, the comparison of the in-situ Raman spectra of all the samples revealed the most pronounced O-O stretching vibrations (ν_*O2_ and ν_*OOH_) signals on the MHCS_0.5_, indicating the accelerated reaction rate within the dominant conformation featuring favorable reaction microenvironments.

We present a summary on the functional attributes of the mesoporous hollow carbon sphere nanoreactor in the electrochemical 2e^-^ ORR. The mesoporous channels facilitate fluid acceleration to transport the generated H_2_O_2_ into the bulk solution, decreasing the likelihood of its further electro-reduction on the catalyst surface^45^. Besides, the accelerated electrolyte flow rapidly pumps substrates to enrich O_2_. Additionally, the accelerated reaction rate and favorable hollow configuration promote localized pH elevation, providing a suitable microenvironment for the 2e^-^ ORR. FES, experimental results, and in-situ Raman spectroscopy verify these functions of the MHNs based on engineered carbon spheres, which can be compared to certain structural functions of cells: the mesoporous shell of the MHNs is analogous to the cell membrane and effectively controls the entry and exit of substrates and products; while the hollow interior of the MHNs, akin to the cytoplasm within cells, furnishes a conducive internal microenvironment for the reaction. Whilst our example may lack the intricate and sophisticated functional units found in biology, it nonetheless serves as a source of inspiration for the construction of nanoreactors through biomimicry.

## Discussion

In summary, this work explored the catalytic functionalities of carbon-based MHNs. In detail, we engineered the interiors of mesoporous carbon spheres, drawing inspiration from FES for fluid diffusion, thereby constructing a series of model nanoreactors. By utilizing electrochemical 2e^-^ ORR as a probe reaction, we provide evidence of the enhanced diffusion, reactant enrichment, and microenvironmental modulation facilitated by the mesoporous hollow architectures. These catalytic functionalities are closely linked to the structural parameters of MHNs, emphasizing the importance of controlling the nanoreactor’s microstructure for targeted reactions. With the catalytic functionalities of the nanoreactor, the optimized MHCS_0.5_ catalysts demonstrate impressive activity and selectivity in H_2_O_2_ electrosynthesis under both alkaline and neutral conditions. Moreover, the generation of medical-grade H_2_O_2_ disinfectant in a flow cell device substantiates the significant practical potential of this nanoreactor engineering. Overall, this study showcases the successful implementation of nanoreactor engineering in screening catalysts with functional orientation, paving the way for a wide range of nanoreactor applications.

## Methods

### Synthesis of mesoporous carbon sphere nanoreactors with different internal structures

In a typical synthesis, a solution of 65 mL of ethanol and 15 mL of H_2_O was mixed with 2.5 mL of NH_3_·H_2_O and 0.8 mL of formaldehyde at room temperature. Subsequently, 0.6 g of resorcinol and 6 mL of TPOS were added to the mixed solution under stirring. The reaction was stirred for 24 h at room temperature, and the products were obtained by centrifugation, followed by washing with water and ethanol, and drying in an oven. The as-obtained products were subjected to pyrolysis at a temperature of 800 °C for 2 h in N_2_ with a heating rate of 2 °C min^−1^, followed by removal of the silica template through etching with HF (5 wt%). The internal structure of the carbon spheres is determined by the temporal order of addition of resorcinol and TPOS. MHCS_0.1_, MHCS_0.5_, MHCS_0.7_, and MCS_−0.5_ were synthesized by adding resorcinol and TPOS at intervals of −20, 0, 20, and 80 min, respectively. CS were prepared without the addition of TPOS. The reduction of MHCS_0.5_ was conducted in a tube furnace at 800 °C for 2 h under a mixed H_2_/N_2_ atmosphere, resulting in the product denoted as R-MHCS_0.5_.

### Material characterizations

Scanning electron microscopy (SEM) images were acquired using a scanning microscope (SU8230, Japan). Transmission electron microscopy (TEM) was performed with Tecnai G2 F30 (America) and JEM-2100 (Japan). Powder X-ray diffraction data were obtained using a Rigaku D/Max2500PC diffractometer with Cu Kα radiation (λ = 1.5418 Å) across the 2θ range of 5−80°, with a scan speed of 5° min^−1^ at room temperature. Raman measurements were conducted using a spectrometer (NanoWizard Ultra Speed & inVia Raman) with a laser having an excitation wavelength of 532 nm. Nitrogen sorption isotherms were recorded at 77 K (Tristar 3020, USA). X-ray photoelectron spectroscopy (XPS) data were collected with a monochromatic Al source (KRATOS, Axis Ultra). Electron paramagnetic resonance (EPR) measurements were carried out using a Bruker ECS-EMX X-band EPR spectrometer at room temperature.

### Electrochemical measurements

All electrochemical measurements were conducted using an electrochemical workstation (PARSTAT 3000A-DX) with a three-electrode cell at room temperature. An Ag/AgCl electrode was used as reference electrode and equipped with a salt bridge to eliminate liquid boundary potentials and prevent cross-contamination between the solution of the study system and the reference electrode system. Additionally, a Pt sheet was employed as the counter electrode, and a catalyst-loaded rotating ring-disk electrode (RRDE) functioned as the working electrode. The RRDE assembly included a glassy carbon rotation disk electrode (disk area: 0.247 cm^2^) and a Pt ring (ring area: 0.186 cm^2^) with a collection efficiency (*N*) of 0.37.

In the preparation of the catalyst ink, 5.0 mg of the electrocatalyst was dispersed in 950 μL of ethanol and 50 μL of a 5 wt% Nafion solution. After ultrasonic treatment for 30 min, 5.0 μL of the catalyst ink was drop-cast onto the disk electrode.

The electrochemical measurements were conducted at room temperature in O_2_-saturated 0.1 M KOH aqueous solution (pH 13) and 0.1 M PBS (pH 7). The working electrode rotated at a constant speed of 1600 rpm during the tests. All potentials were converted to reference the reversible hydrogen electrode (RHE) using the following equation:

1 $${E}_{{{{{{\rm{RHE}}}}}}}={E}_{{{{{{\rm{Ag}}}}}}/{{{{{\rm{AgCl}}}}}}}+0.197+0.059\times {{{{{\rm{p}}}}}}{{{{{\rm{H}}}}}}$$

Stable linear sweep voltammetry (LSV) was performed at a scan rate of 10 mV s^−1^ in electrolytes saturated with N_2_ and O_2_. To ensure the complete oxidation of H_2_O_2_, the ring electrode was maintained at a constant potential of 1.2 V vs RHE. The selectivity of H_2_O_2_ and the electron transfer number (*n*) were calculated were calculated based on the disk current (*I*_d_) and ring current (*I*_r_) as following equations:

2 $${{{{{{\rm{H}}}}}}}_{2}{{{{{{\rm{O}}}}}}}_{2}\%=\frac{200\times {I}_{{{{{\rm{r}}}}}}}{\left(N\times {{{\mbox{|}}}I}_{{{{{\rm{d}}}}}}{{\mbox{|}}}\right)+{I}_{{{{{\rm{r}}}}}}}$$

3 $$n=\frac{4\times {I}_{{{{{\rm{d}}}}}}}{{{\mbox{|}}}{I}_{{{{{\rm{d}}}}}}{{\mbox{|}}}+{I}_{{{{{\rm{r}}}}}}/N}$$

### Measurement of electrochemically active surface area

The electrochemically active surface area (ECSA) was determined using the double-layer capacitance method. Constant potential cyclic voltammetry (CV) scans were performed in the double-layer region, ranging from −0.06 to 0.04 V vs Ag/AgCl, with scan rates of 20, 40, 60, 80, and 100 mV s^−1^. Plotting the sweep speed as the abscissa and (*j*_a_ – *j*_c_) as the ordinate, the slope value was calculated to represent the double-layer capacitance (*C*_dl_), which is linearly related to ECSA. ECSA was determined by calculating *C*_dl_ using the following equations:

4 $${{{{{\rm{E}}}}}}{{{{{\rm{CSA}}}}}}=\frac{{C}_{{{{{\rm{dl}}}}}}}{{C}_{{{{{\rm{s}}}}}}}\times {A}_{{{{{{\rm{GCE}}}}}}}$$

Where the specific capacitance (*C*_s_) for the electrode surface, typically falling within the range of 20 to 60 μF cm^−2^. Specifically, we adopted a value of 40 μF cm^−2^. *A*_GCE_ denotes the electrode surface area.

### Bulk electrolysis

The bulk electrosynthesis of H_2_O_2_ in 0.1 M KOH was conducted using a customized H-cell electrolyzer to further investigate the practical performance of H_2_O_2_ electrosynthesis. The working electrode employed was a carbon-paper electrode (1 × 1 cm^2^) coated with MHCS_0.5_ (0.25 mg cm^−2^). As for the counter and reference electrodes, a Pt electrode and an Ag/AgCl (saturated KCl) electrode were utilized, respectively. Chambers were separated by a Nafion membrane. The electrolyte consisted of 30 mL O_2_-saturated 0.1 M KOH aqueous solution.

### Direct detection of the local pH changes on electrode

For the indirect observation of changes in local pH, the MHCS_0.5_ catalyst was employed to facilitate the 2e^-^ ORR process for 10 min in the electrolytic cell (0.1 M PBS, pH = 7, 0.4 V vs RHE). Subsequently, the applied voltage was switched off, and the working electrode was transferred to 5 mL of aqueous solution and rinsed. After repeating these two steps 10 times, the pH value was measured in the reagent bottle using a pH meter. To ensure experimental accuracy, this parallel experiment was conducted 5 times.

For a direct observation of pH elevation on the electrode surface, we monitored the color change on the MHCS_0.5_ electrode surface during the 2e^-^ ORR at 0.1 V vs RHE. The reaction was conducted in a H-type electrolytic cell using 0.1 M K_2_SO_4_ containing 1 mg of phenolphthalein as the electrolyte for visualization.

### H_2_O_2_ yield in the flow cell

The electrochemical synthesis of H_2_O_2_ via ORR was carried out using a two-compartment flow cell setup separated by a Nafion membrane. The cathode assembly in the gas diffusion layer was created by applying the MHCS_0.5_ catalyst ink onto the carbon paper, with an actual working area of 1 cm^2^ and a catalyst loading of 0.25 mg cm^−2^. Ag/AgCl was employed as the reference electrode, and IrO_2_ served as the anode. In the flow cell test, a 0.1 M KOH/0.1 M PBS electrolyte (40 mL) was circulated through each compartment at a flow rate of 6 mL min^−1^, while the cathode received a continuous supply of O_2_ at a rate of 20 mL min^−1^.

### H_2_O_2_ concentration measurement

The amount of H_2_O_2_ was determined using a conventional cerium sulfate titration method, following the equation:

5 $$2{{{{{\rm{Ce}}}}}}^{4+}+{{{{{\rm{H}}}}}}_2{{{{{\rm{O}}}}}}_2=2{{{{{\rm{Ce}}}}}}^{3+}+2{{{{{\rm{H}}}}}}^{+}+{{{{{\rm{O}}}}}}_{2}$$

In this method, yellow Ce^4+^ is reduced by H_2_O_2_ to colorless Ce^3+^. A 0.5 mmol L^−1^ Ce(SO_4_)_2_ solution was typically prepared by dissolving 16.6 mg Ce(SO_4_)_2_ in 100 mL of 0.50 mol L^−1^ H_2_SO_4_ acid solution. The calibration curve was established by linear fitting of absorbance values at a wavelength of 320 nm for known concentrations of 0.01, 0.02, 0.05, 0.1, 0.2, 0.3, 0.4, and 0.5 mmol L^−1^ Ce^4+^. Using this calibration curve, the concentration of reduced Ce^4+^ in the sample solution was determined by measuring UV–vis absorption intensity (Supplementary Fig. 50). Finally, the H_2_O_2_ yield was calculated based on the known concentration of reduced Ce^4+^. The faradaic efficiency (FE) for H_2_O_2_ generation is calculated as the following equation:

6 $${{{{{\rm{FE}}}}}}\,(\%)=\frac{100\%\times {{{{{\rm{mole}}}}}}\,{{{{{\rm{of}}}}}} \,{{{{{\rm{generated}}}}}}\,{{{{{\rm{H}}}}}}_{2}{{{{{\rm{O}}}}}}_{2}\times 2\times 96485}{{{{{{\rm{total}}}}}}\,{{{{{\rm{consumed}}}}}}\,{{{{{\rm{charge}}}}}}}$$

### Finite element simulation

A simulation was conducted using COMSOL Multiphysics software to investigate the relationship between electrolyte flow and mass transport in the mesoporous hollow nanoreactor (MHN) model, accounting for the convective and diffusive effects of O_2_, H_2_O, and OH^-^. Both 3D and 2D models were employed to simulate the flow rate and material distribution in the MHNs, respectively.

To model the synthesized MHCS_x_, MHNs were constructed uniformly with a particle radius of 150 nm and a mesopore channel of approximately 20 nm, with the proportion of the hollow component as the sole variable. This approach effectively illustrates the impact of various *d*/*r* ratios on fluid transport at a standardized particle size, optimizing computational efficiency without compromising accuracy. Additionally, models with different pore sizes were simulated and compared. For simplicity, deformation was disregarded, and the model was assumed to be in a fixed position. Two 10 nm spaces were established on the left and right sides of the mesoporous channel to simulate the inflow and outflow of substances in the nonporous structure. The simulation model employed axisymmetric geometry to calculate fluid velocity and pressure in the mesoporous and hollow regions, as well as the concentration distribution of reactant substances. In this model, the left and right sides were designated as the inlet and outlet of the electrolyte, respectively, with mass transfer driven by fluid flow and concentration diffusion. The governing equations are as follows:

7 $$\rho ({{{{{\bf{u}}}}}}\cdot \nabla ){{{{{\bf{u}}}}}}=\nabla \cdot [-{{{{{\rm{p}}}}}}{{{{{\bf{I}}}}}}+{{{{{\bf{K}}}}}}]+{{{{{\bf{F}}}}}}$$

8 $$\rho \nabla \cdot {{{{{\bf{u}}}}}}=0$$

9 $${{{{{\bf{K}}}}}}=\mu (\nabla {{{{{\bf{u}}}}}}+{(\nabla {{{{{\bf{u}}}}}})}^{{{{{{\rm{T}}}}}}})$$

10 $$\nabla \,{\cdot } \, {{{{{{\bf{J}}}}}}}_{i}+{{{{{\bf{u}}}}}} \, {\cdot } \, {\nabla }_{ci}={R}_{i}$$

11 $${{{{{{\bf{J}}}}}}}_{i}={-D}_{i}{\nabla c}_{i}$$

where the dependent variables are velocity (**u**), pressure (*p*), and concentration (*c*_*i*_). ρ is the fluid density, *μ* is the fluid viscosity, *D*_*i*_ is the diffusion coefficient of substance i, and *R*_*i*_ is the reaction rate. For the 3D/2D model of MHNs, electrolyte ingress occurs from the left, traverses the model region, and egresses from the right. To emulate the flow induced by solution rotation, the inlet velocity was set to 0.235 m s^−1^, with a zero-pressure boundary condition at the outlet. Given the low Reynolds number regime (Re ~ 10^−4^) resulting from the small size of MHCS_x_, the “Laminar Flow” module was chosen to simulate the flow field. At the model’s inlet boundary, a solution saturated with O_2_ was set, having a concentration of 21.4 mg L^−1^ (0.66875 mol m^3^) at the top inflow boundary. The outflow boundary was configured with a diffusion flux set to 0. The model’s surface was designated as the location where the reaction occurs, and O_2_ is consumed for conversion. Throughout the simulation, we examined the impacts of geometric dimensions, such as mesopore diameter and spacing, on O_2_ and H_2_O_2_ concentrations. The geometric parameters required for the simulation were derived from experimental data, including electron microscope images.

### Supplementary information

Supplementary Information

Peer Review File

Description of Additional Supplementary Files

Supplementary Movie 1

## Data Availability

The main data supporting the findings of this study are available within the main text and the Supplementary Information file. The main data generated in this study have been deposited in the figshare database under the accession code 10.6084/m9.figshare.24935178.
