# Supplementary material for: Mesoporous carbon spheres with programmable interiors as efficient nanoreactors for H2O2 electrosynthesis
Source: Nat Commun. 2024 Feb 1;15:983. doi: 10.1038/s41467-024-45243-w (PMC10834542; doi:10.1038/s41467-024-45243-w)
Supplement: Supplementary file 1 — Supplementary Information [file 41467_2024_45243_MOESM1_ESM.pdf]

Supplementary Information

Mesoporous Carbon Spheres with Programmable Interiors as Efficient Nanoreactors for H_2O_2 Electrosynthesis

Qiang Tian,[1,2] *Lingyan Jing,*[2,3]* *Hongnan Du,*[4] *Yunchao Yin,*[1,2] *Xiaolei Cheng,*[1] *Jiaxin Xu,*[1] *Junyu Chen,*[1] *Zhuoxin Liu,*[1] *Jiayu Wan,*[5] *Jian Liu,*[4] *Jinlong Yang*[1]*

1 Shenzhen Key Laboratory of Energy Electrocatalytic Materials, Guangdong Research Center for Interfacial Engineering of Functional Materials, College of Materials Science and Engineering, Shenzhen University, Shenzhen 518060, China.

2 College of Physics and Optoelectronic Engineering, Shenzhen University, Shenzhen 518060, China.

3 College of Chemistry and Environmental Engineering, Shenzhen University, Shenzhen 518060, China.

4 State Key Laboratory of Catalysis, Dalian Institute of Chemical Physics, Chinese Academy of Sciences, 457 Zhongshan Road, Dalian 116023, China.

5 Global Institute of Future Technology, Shanghai Jiaotong University, Shanghai 200240, China.

E-mail: lingyan.jing@foxmail.com (**L. Jing**); yangjl18@szu.edu.cn (**J. Yang**).

Supplementary Tables

Supplementary Table. 1 Geometric values of $MHCS_x$ and CS.

Sample / Parameter	*CS*	*$MHCS_{0.7}$*	*$MHCS_{0.5}$*	*$MHCS_{0.1}$*	*$MCS_{-0.5}$*
Average radius of particles	135 nm	160 nm	210 nm	155 nm	190 nm
Average thickness of mesoporous shells	—	50 nm	105 nm	140 nm	85 nm
Average radius of hollows	—	110 nm	105 nm	15 nm	—
Average radius of the cores	—	—	—	—	110 nm
Shell thickness/ particle radius	—	~31%	~50%	~90%	~45%
Hollow radius of /particle radius	—	~70%	~50%	~10	~-55%

Supplementary Table. 2 Elemental contents of C and O according to XPS spectra and relative content of different O configurations for $MHCS_x$.

**Sample**	**Atomic content (at.%)**		**Relative content (%)**	
	C	O	C=O	C–O
$MHCS_{0.7}$	90.6	9.4	59.03	40.97
$MHCS_{0.5}$	90.7	9.3	58.92	41.08
$MHCS_{0.1}$	90.7	9.3	59.07	40.93
$MCS_{-0.5}$	90.4	9.6	59.04	40.96

Supplementary Table. 3 Comparison of electrocatalytic H_2O_2 production performance by RRDE technique in 0.1 M KOH electrolyte (pH = 13) of recently reported electrocatalysts.

Catalyst	Onset potential (V_{RHE})	H_2O_2 selectivity (%)	Potential (V_{RHE})	Ref.
$MHCS_{0.5}$	0.85	> 95	0.3-0.8	This work
Co-N/HPC	0.8	~ 95	0.25-0.65	1
CQD	0.8	> 90<br>> 95	0.25-0.65<br>0.45-0.65	2
O-HGr	0.78	> 90<br>~ 95	0.2-0.75<br>0.45-0.75	3
CF	0.81	~ 90	0.55-0.63	4
N-FLG-8	0.76	> 95	0.35-0.7	5
$OCNS_{800}$	0.82	~ 90	0.5-0.75	6
NiB_2	0.68	> 90<br>~ 95	0.2-0.6<br>0.4-0.6	7
O-GOMC	0.8	~ 90	0.2-0.75	8
Mo_1/OSG-H	0.78	~ 95	0.4-0.7	9
Co-N-C	0.78	70-80	0.1-0.8	10
Ni-N_2O_2/C	0.7	> 90<br>~ 95	0.1-0.5<br>0.4-0.5	11
Cu-Pb	0.7	> 90<br>~ 95	0.4-0.57<br>0.52-0.57	12
Fe-CNT	0.82	~ 90	0.55-0.75	13
ZnO_3C	0.73	~ 80	0.5-0.7	14
Co–POC–O	0.82	~ 80	0.55-0.8	15
Pd@$Au_{0.95}Pd_{0.05}$	0.7	~ 90	0.4-0.7	16
Bi2Te3	0.76	> 95	0.2-0.6	17
GLC	0.8	~ 90	0.4-0.6	18
CoNPs@N/C	0.83	~ 90	0.1-0.6	19
OCG-800	0.78	> 95	0.3-0.7	20
CB-Plasma	0.8	> 90<br>~ 95	0.4-0.7<br>0.5-0.6	21

In SAs/NSBC	0.8	> 90	0.4-0.7	22
P-NMG-10	0.78	80-90	0-0.7	23
N,S-TCNTs	0.78	~ 90	0.2-0.8	24
Fe_{SA}-NS/C-700	0.75	92	0.3-0.5	25
NBO-G/CNTs	0.8	80-90	0.25-0.75	26
FS-CFs	0.81	~ 85	0.5-0.8	27
In_2O_3/CDs–10	0.76	~ 95	0.4-0.7	28
$Pb(NiWMnNbZrTi)_{1/6}O_3$	0.76	~ 95	0.3-0.7	29
Co-SCD-2	0.78	> 90	0.1-0.6	30
NiPyPC/CN	0.77	~ 90	0.25-0.65	31

Supplementary Table. 4 Comparison of electrocatalytic H_2O_2 production performance by RRDE technique in neutral electrolyte (pH = 7) of recently reported electrocatalysts.

**Catalyst**	**Electrolyte**	**Onset potential (V_{RHE})**	**j_{disk} (mA cm^{-2}) @ 0.2 V_{RHE}**	**Selectivity (%@V_{RHE})**	**Ref.**
$MHCS_{0.5}$	0.1 M PBS	0.60	-2.8	85-90% @ (0.1-0.35) >90% @ (0.30-0.50)	This work
B-C	0.1 M Na_2SO_4	0.45	-2.1	70-80% @ (0.1-0.35)	32
O-CNTs	0.1 M PBS	0.5	-0.24	80-90% @ (0.2-0.5)	33
MBC-2	0.5 M Na_2SO_4	0.4	-1.6	80-90% @ (0.2-0.4)	34
N-FLG-8	0.1 M Na_2SO_4	0.5	-2.5	70-80% @ (0.1-0.40)	5
Co-N-C	0.1 M PBS	0.62	-2.5	>60% @ (0.3-0.4)	10
h-SnO_2	0.1 M Na_2SO_4	0.45	-2.1	94-99% @ (0-0.40)	35
CoPc-CNT(O)	0.1 M K_2SO_4	0.52	-2.7	>90% @ (0.35-0.55)	36
a-$PdSe_2$ NPs/C	0.1 M Na_2SO_4	0.4	-1.8	>90% @ (0-0.30)	37
Zn_{NP}-O-C	0.1 M PBS	0.57	-0.2	> 90% @ (0-0.40)	38
Fe-CNT	0.1 M PBS	0.53	-2.8	> 90% @ (0.30-0.50)	13
MCNS	0.1 M PBS	0.53	-1.8	> 90% @ (0.13-0.43)	29
O-C(Al)	0.1 M PBS	0.52	-2.3	85-90% @ (0.1-0.45)	30
L-ZnO	0.6 M K_2SO_4	0.38	-0.9	~ 90 @ 0.2	39
ZnO@ZnO_2	0.1 M K_2SO_4	0.42	-2.7	> 90 @ (0-0.4)	40
In SAs/NBSC	0.1 M PBS	0.5	-2.7	> 90 @ (0.1-0.35)	22
ZnCo-ZIF	0.1 M PBS	0.48	-1.8	~ 90 @ (0.05-0.3)	41
O-HGr	0.1 M PBS	0.5	-1.9	~ 91 @ 0.2	3

Supplementary Table. 5 Comparison of electrochemical performance in the flow cell device with recently reported electrocatalysts.

Catalysts	Electrolyte	Condition	H_2O_2 yield (mol g^{-1} h^{-1})	FE (%)	Ref.
$MHCS_{0.5}$	0.1 M KOH	0.1 V vs RHE	17.18	90	This work
$MHCS_{0.5}$	0.1 M PBS	0.1 V vs RHE	12.64	90	This work
FeSA-NS/C-700	0.1 M KOH	0 V vs RHE	4.95	91.4	25
CNB-ZIL 8	0.1 M KOH	-1.4 V (cell voltage)	1.79	80	42
Ni-N_2O_2	0.1 M KOH	70 mA cm^{-2}	5.9	90	11
N-FLG-8	1 M KOH	1.8 V (cell voltage)	9.66	90	5
Co-NC	0.1 M KOH	50 mA cm^{-2}	4.2	42	43
N-O-C-800	0.1 M KOH	0.25 V vs RHE	1.47	65	44
OCNS900	0.1 M KOH	0.2 V vs RHE	0.77	60	6
ZnCo-ZIF	0.1 M KOH	60 mA cm^{-2}	4.3	70	41
ZnCo-ZIF	0.1 M PBS	70 mA cm^{-2}	3.8	72	41
Ni_4-B_1@BNC	1 M KOH	0.2 V vs RHE	0.13	73	45
Sb-NSCF	1 M KOH	0.55 V vs RHE	7.46	80	46
In_2O_3/CDs–10	0.1 M KOH	0.5 V vs RHE	4.5	93	28
CoPc-OCNT	1 M KOH	300 mA cm^{-2}	11.527	96	39
h-SnO_2	1 M KOH	0.5 V vs RHE	3.18	/	35
h-SnO_2	1.0 M Na_2SO_4	0 V vs RHE	3.8	72	35
ZnO@ZnO_2	0.1 M K_2SO_4	0 .1 V vs RHE	5.47	95	40
In SAs/NSBC	0.1 M Na_2SO_4	90 mA cm^{-2}	6.71	75	22
In SAs/NSBC	0.1 M KOH	90 mA cm^{-2}	6.49	78	22
Co-N-C	0.5 M NaCl	50 mA cm^{-2}	4.5	75	47
NBO-G/CNTs	0.1 M KOH	50 mA cm^{-2}	0.71	81	26
PBT	1.0 M KOH	100 mA cm^{-2}	3.13	96	48
PANI/CDs-Co-2	0.1 M KOH	0 V vs RHE	3.5	86	49
NiB_2	0.1 M KOH	0.4 V vs RHE	4.75	90	7

Supplementary Figures

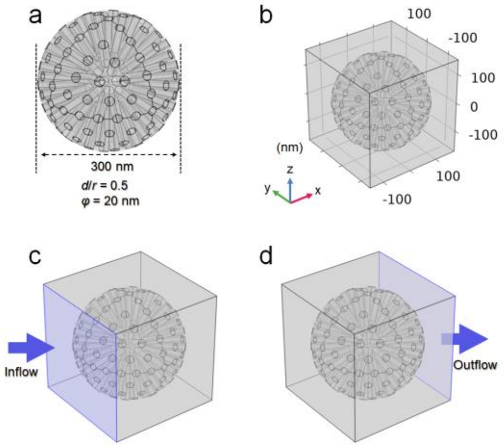

Supplementary Fig. 1 a Mesoporous hollow carbon sphere model (*d*/*r* = 0.5, *r* = 150 nm, *φ* = 20 nm). **b** Model of mesoporous hollow carbon spheres in a three-dimensional fluid field (320 nm × 320 nm × 320 nm). **c** Schematic diagram of the fluid field inlet. **d** Schematic diagram of the fluid field outlet.

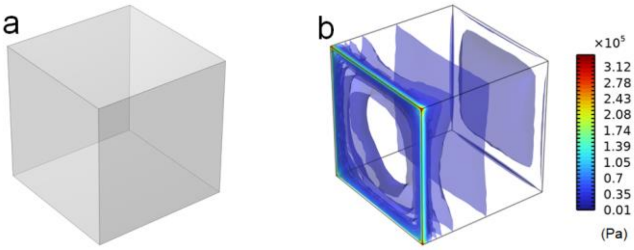

Supplementary Fig. 2 a Schematic diagram of the fluid field without any model and **b** the equivalent surface of pressure distribution.

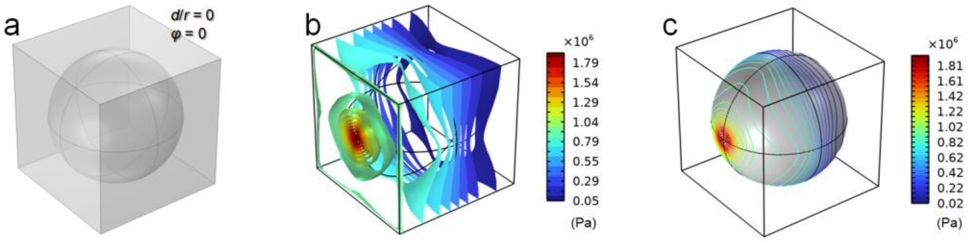

Supplementary Fig. 3 a Schematic diagram of the fluid field with a non-porous sphere model (*d*/*r* = 0, *r* = 150 nm, *φ* = 0) inside. **b** Equivalent surface of pressure distribution in the fluid field with a non-porous sphere model (*d*/*r* = 0, *r* = 150 nm, *φ* = 0) inside. **c** Contours of the pressure distribution on the non-porous sphere model (*d*/*r* = 0, *r* = 150 nm, *φ* = 0).

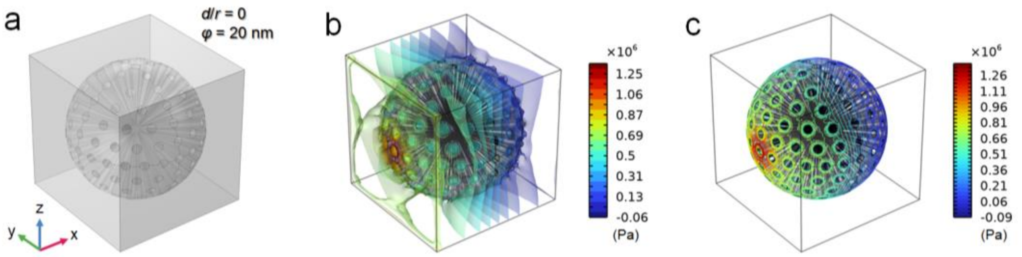

Supplementary Fig. 4 **a** Schematic diagram of the fluid field with a mesoporous porous sphere model (d/r = 0, r = 150 nm, φ = 20 nm) inside. **b** Equivalent surface of pressure distribution in the fluid field with a mesoporous sphere model (d/r = 0, r = 150 nm, φ = 20 nm) inside. **c** Contours of the pressure distribution on the mesoporous sphere model (d/r = 0, r = 150 nm, φ = 20 nm).

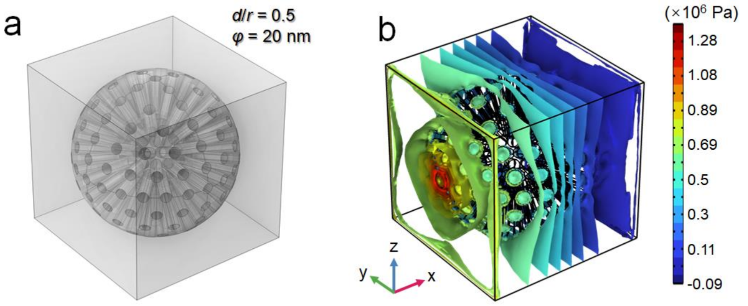

Supplementary Fig. 5 **a** Schematic diagram of the fluid field with a mesoporous porous sphere model (d/r = 0.5, r = 150 nm, φ = 20 nm) inside. **b** Equivalent surface of pressure distribution in the fluid field with a mesoporous sphere model (d/r = 0.5, r = 150 nm, φ = 20 nm) inside.

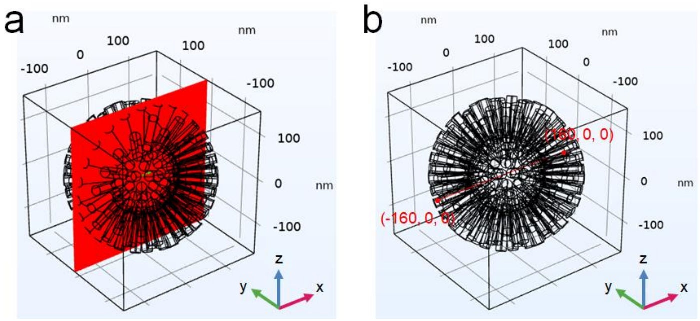

Supplementary Fig. 6 **a** Schematic diagram of a cross section (y = 0) in fluid field space. **b** Schematic diagram of a truncated line (-160, 0, 0 →160, 0, 0) in fluid field space.

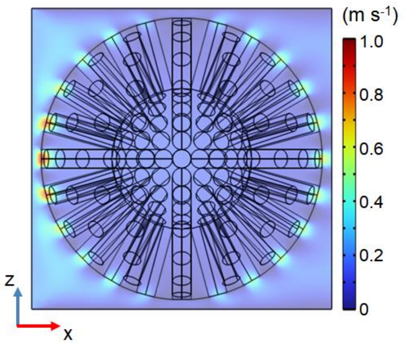

Supplementary Fig. 7 Cross-section (y = 0) of the spatial flow velocity distribution.

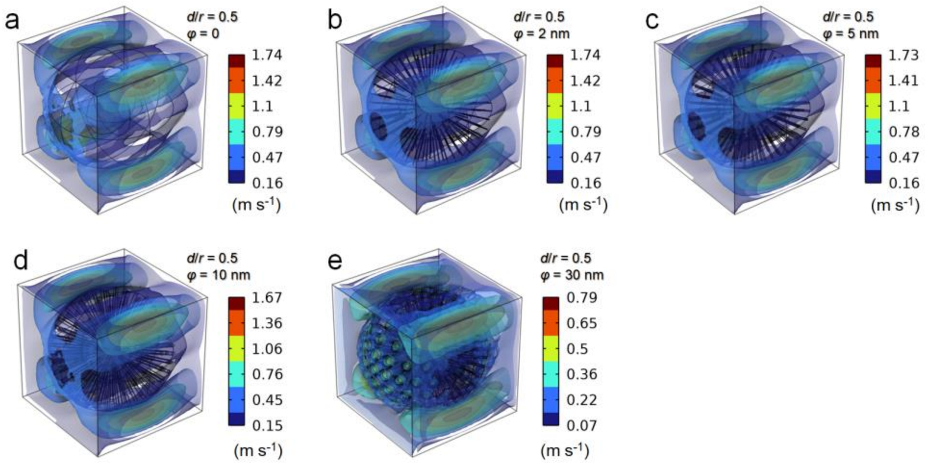

Supplementary Fig. 8 Spatial distribution of fluid velocity in the hollow porous sphere models (*d*/*r* = 0.5) with different pore sizes: **a** 0, **b** 2 nm, **c** 5 nm, **d** 10 nm, and **e** 30 nm.

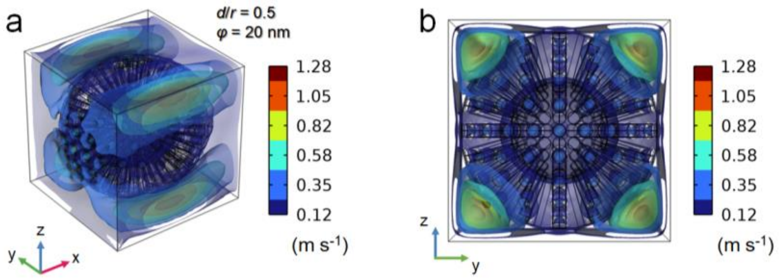

Supplementary Fig. 9 a Spatial distribution of fluid velocity in the hollow mesoporous sphere model ($d/r = 0.5$, $r = 150$ nm, $\varphi = 20$ nm) and **b** the corresponding side view.

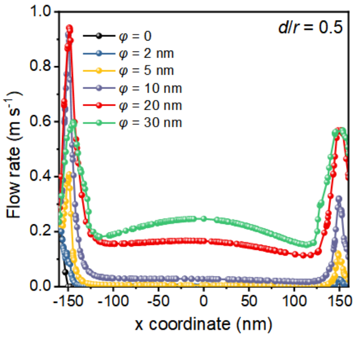

Supplementary Fig. 10 Fluid velocity distribution across mesoporous hollow carbon sphere models (d/r = 0.5) with varying pore sizes (0, 2 nm, 5 nm, 10 nm, 20 nm, and 30 nm).

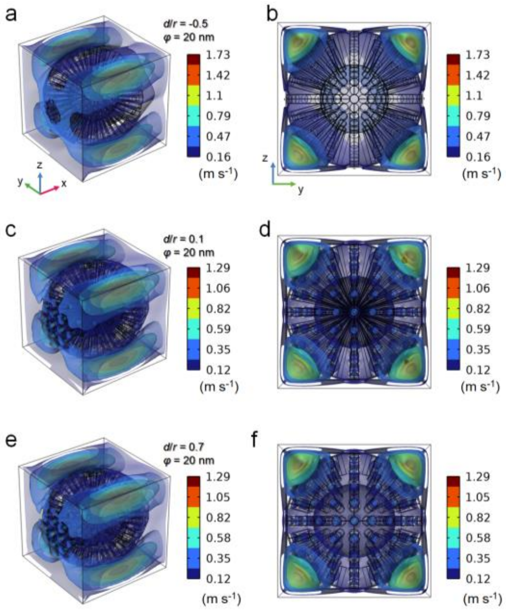

Supplementary Fig. 11 Spatial distribution of fluid velocity in the hollow mesoporous sphere models (φ = 20 nm) with different hollow size: **a** d/r = -0.5, **c** d/r = 0.1, and **e** d/r = 0.7. **b**, **d**, and **f** are the side views of **a**, **c** and **e** respectively.

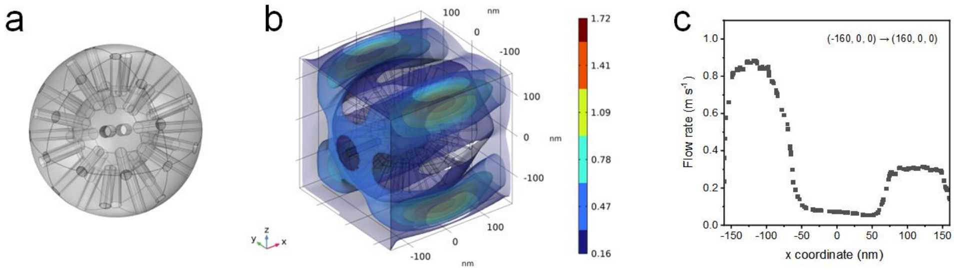

Supplementary Fig. 12 a Schematic modeling of mesoporous hollow sphere (d/r = 0.5, r = 150 nm, φ = 20 nm) with reduced mesopore density. **b** Spatial distribution of fluid velocity in the hollow mesoporous sphere model with reduced mesopore density. **c** Fluid velocity distribution across mesoporous hollow carbon sphere model with reduced mesopore density.

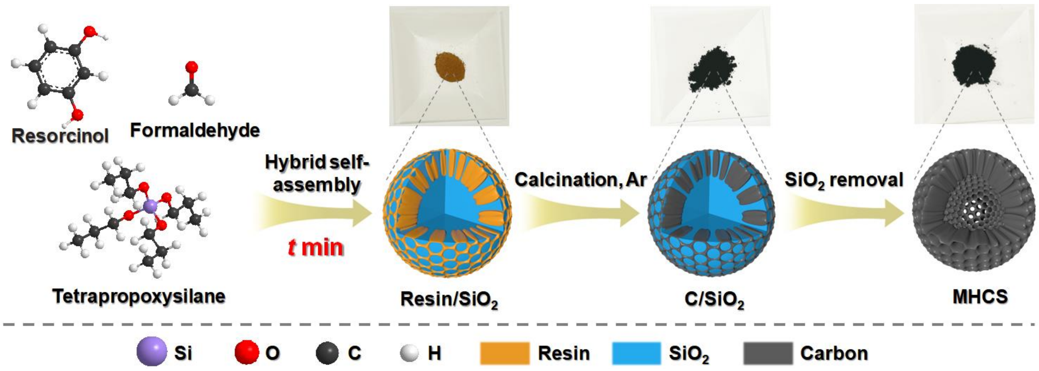

Supplementary Fig. 13 Schematic diagram of sequential organic-inorganic hybridization co-assembly for the synthesis of MHCS.

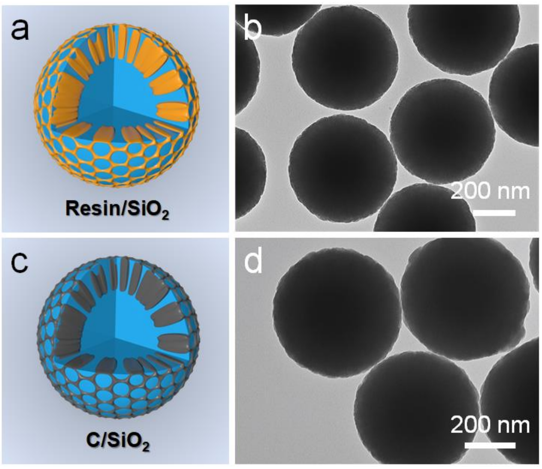

Supplementary Fig. 14 a Structural model and **b** TEM image of resin/SiO_2. **c** Structural model and **d** TEM image of C/SiO_2.

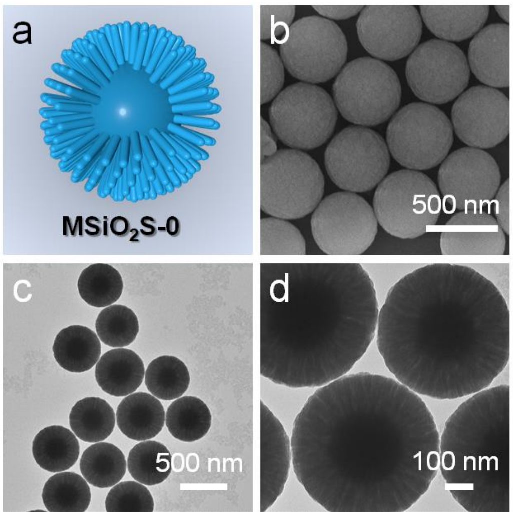

Supplementary Fig. 15 a Structural model, **b** SEM image, and **c**, **d** TEM images of $MSiO_2S$ at $t = 0$.

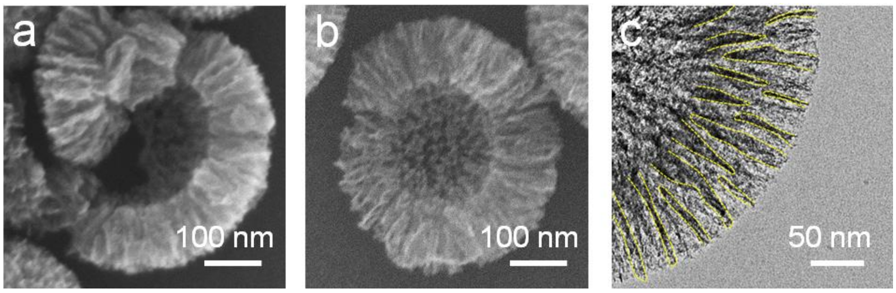

Supplementary Fig. 16 a, b SEM images of crushed $MHCS_{0.5}$ particles. **c** Partially enlarged TEM image of $MHCS_{0.5}$.

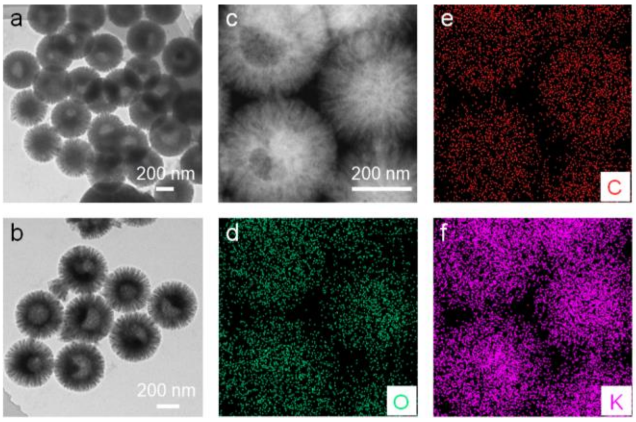

Supplementary Fig. 17 a, b TEM images of $MHCS_{0.5}$ samples dried directly after 1-hour electrolysis in 0.5 M KCl electrolyte. **c-f** HAADF-STEM image, and the corresponding elemental mapping images of $MHCS_{0.5}$ dried directly after 1-hour electrolysis in 0.5 M KCl electrolyte.

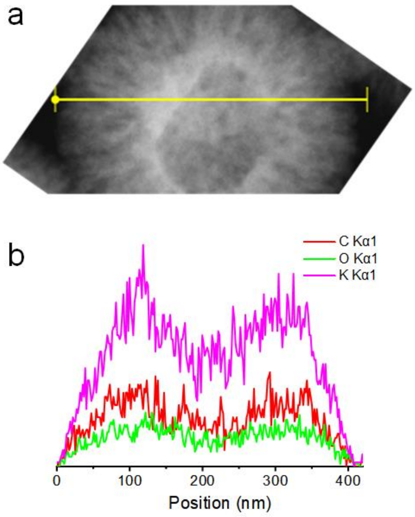

Supplementary Fig. 18 a Linear scan positions on $MHCS_{0.5}$ sample dried directly after 1-hour electrolysis in 0.5 M KCl electrolyte, and **b** the corresponding linear scans of C, O, and K elements.

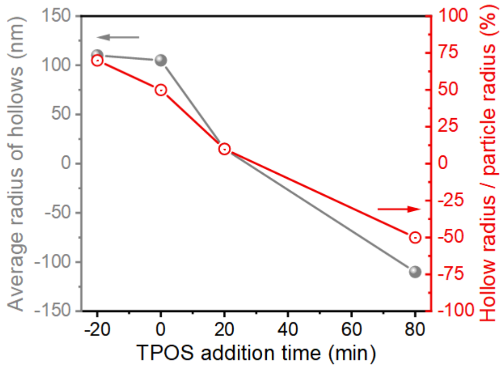

Supplementary Fig. 19 Correlation between the addition time of TPOS and the size of the synthesized hollow as well as the proportion of the hollow.

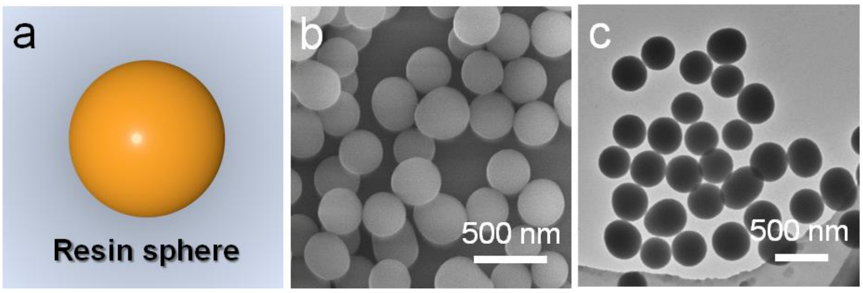

Supplementary Fig. 20 **a** Structural model, **b** SEM image, and **c** TEM image of resin sphere.

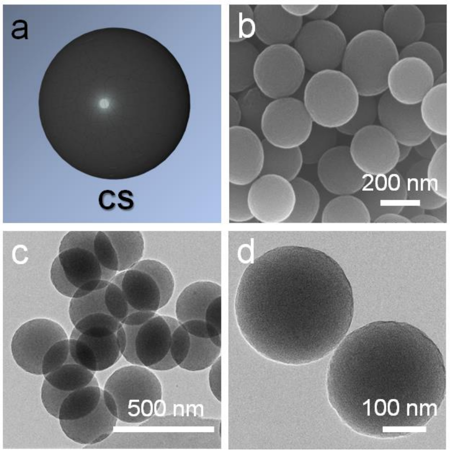

Supplementary Fig. 21 **a** Structural model, **b** SEM image, and **c**, **d** TEM images of CS.

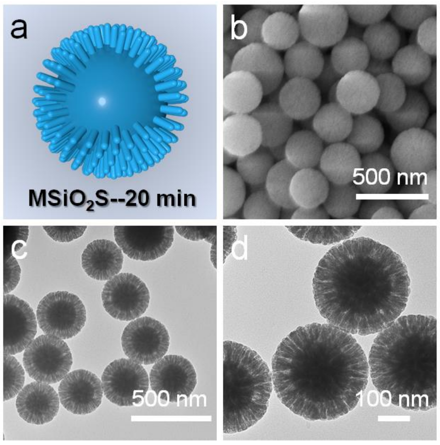

Supplementary Fig. 22 a Structural model, **b** SEM image, and **c**, **d** TEM images of $MSiO_2S$--20 min.

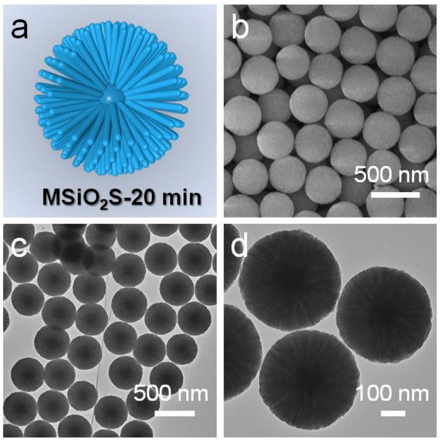

Supplementary Fig. 23 a Structural model, **b** SEM image, and **c**, **d** TEM images of $MSiO_2S$-20 min.

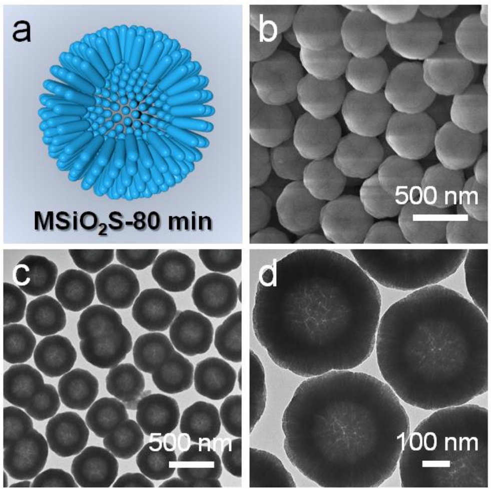

Supplementary Fig. 24 a Structural model, **b** SEM image, and **c**, **d** TEM images of $MSiO_2S$-80 min.

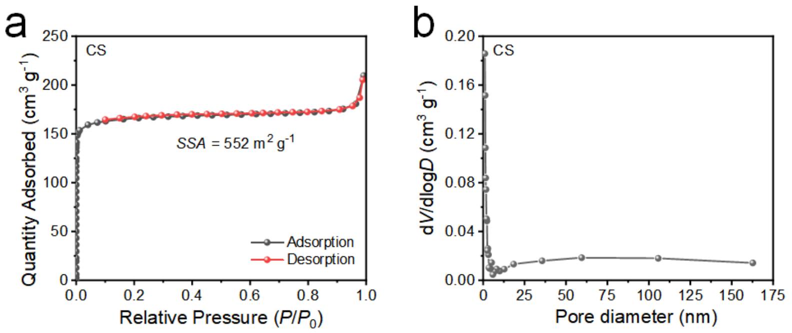

Supplementary Fig. 25 a Nitrogen adsorption-desorption isotherms and **b** the corresponding pore size distribution curves of CS.

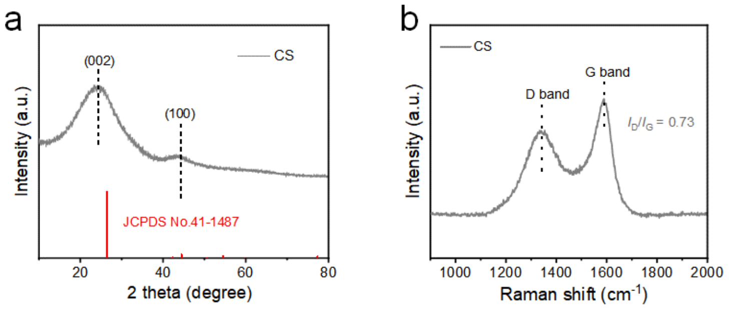

Supplementary Fig. 26 a XRD patterns and **b** Raman spectra of CS.

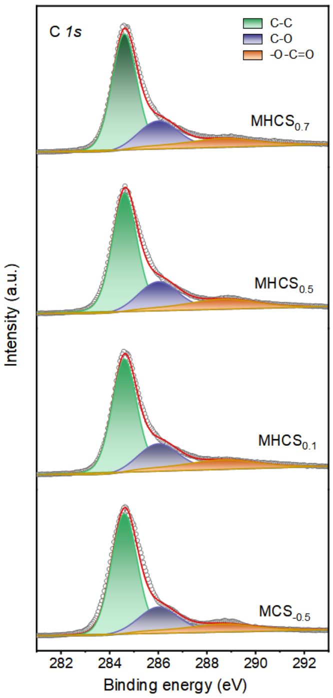

Supplementary Fig. 27 High-resolution C *1s* XPS spectra of $MHCS_x$.

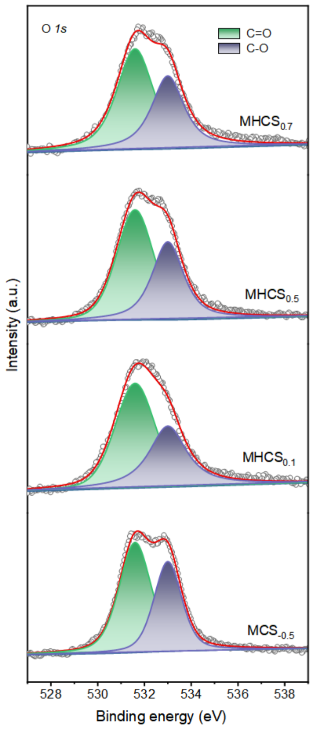

Supplementary Fig. 28 High-resolution O *1s* XPS spectra of $MHCS_x$.

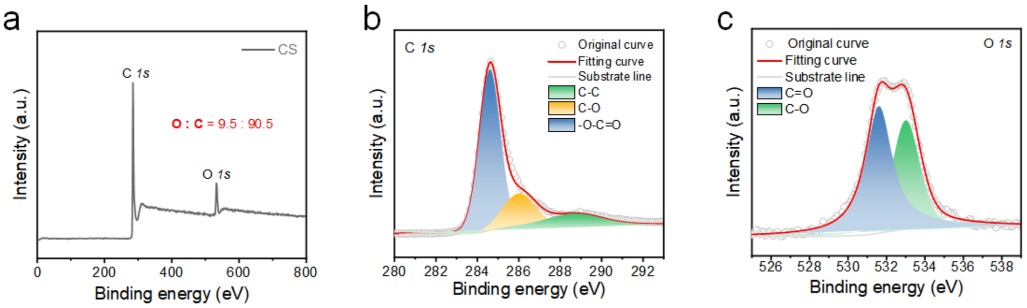

Supplementary Fig. 29 a XPS survey spectra, **b** high-resolution C *1s* XPS spectra and **c** high-resolution O *1s* XPS spectra for CS.

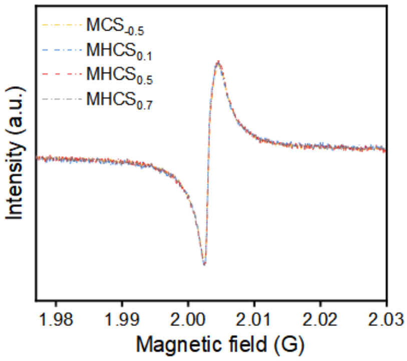

Supplementary Fig. 30 EPR spectra of $MHCS_x$. The obtained $MHCS_x$ samples were analyzed using EPR spectra to evaluate the presence of unpaired electrons, demonstrating comparable signal intensities to suggest similar levels of carbon defects or edges.

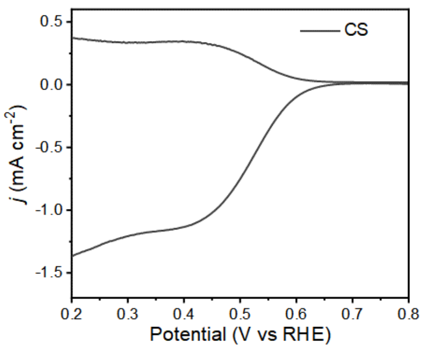

Supplementary Fig. 31 LSV curves of CS recorded at 1600 rpm in O_2-saturated 0.1 M KOH (pH = 13).

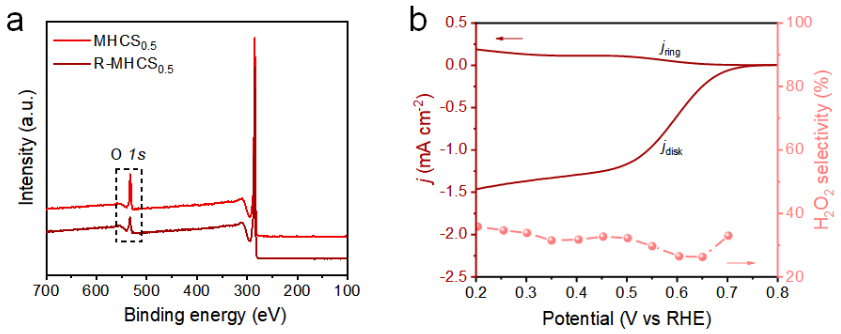

Supplementary Fig. 32 a XPS survey spectrum of R-MHCS$_{0.5}$. **b** LSV curves of R-MHCS$_{0.5}$ recorded at 1600 rpm in an O_2-saturated 0.1 M KOH and the corresponding H_2O_2 selectivity.

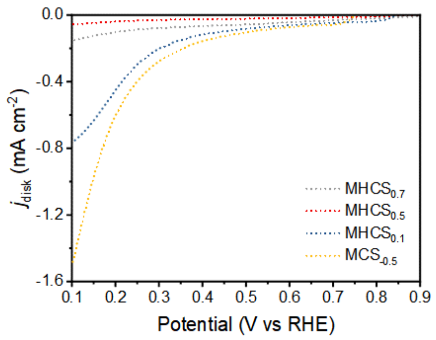

Supplementary Fig. 33 LSV curves for H_2O_2RR recorded on MHCS$_x$ in N_2-saturated 0.1 M KOH containing 10 mM H_2O_2.

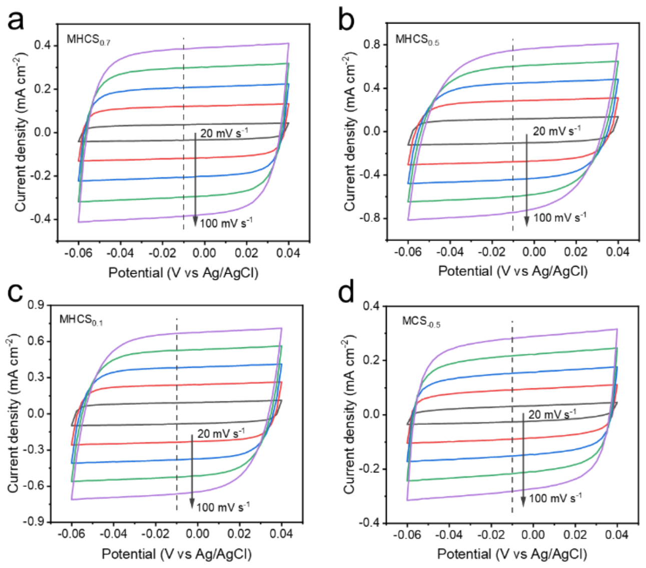

Supplementary Fig. 34 CV curves of **a** $MHCS_{0.7}$, **b** $MHCS_{0.5}$, **c** $MHCS_{0.1}$ and **d** $MCS_{-0.5}$ in the double layer region at scan rates of 20, 40, 60, 80, and 100 mV s^{-1} in 0.1 M KOH (pH = 13) aqueous electrolyte.

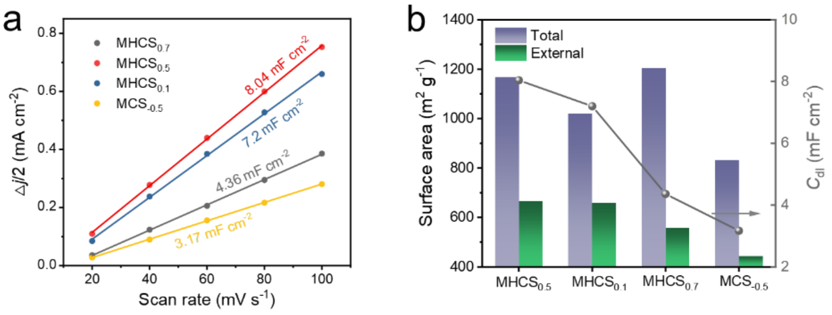

Supplementary Fig. 35 a Capacitance current densities measured at -0.01 V vs Ag/AgCl (saturated KCl) as a function of scan rate. **b**, Total BET surface areas from Nitrogen adsorption-desorption isotherms, external surface areas calculated by the *t*-plot method[50] and double layer capacitance (C_{dl}) for $MHCS_x$. The external surface area refers to the BET surface area excluding the microporous area.

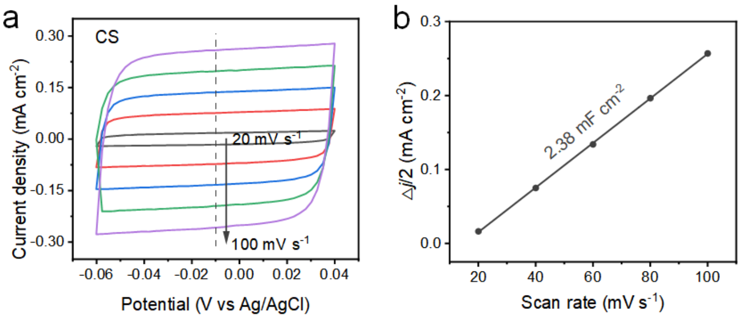

Supplementary Fig. 36 a CV curves of CS in the double layer region at scan rates of 20, 40, 60, 80, and 100 mV s^{-1} in 0.10 M KOH (pH = 13) aqueous electrolyte. **b** Capacitance current densities measured at -0.01 V vs Ag/AgCl (saturated KCl) as a function of scan rate.

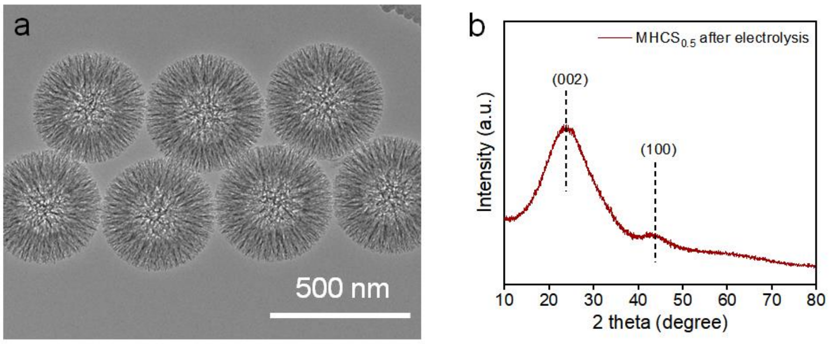

Supplementary Fig. 37 a TEM image and **b** XRD patterns of $MHCS_{0.5}$ after long-term electrolysis.

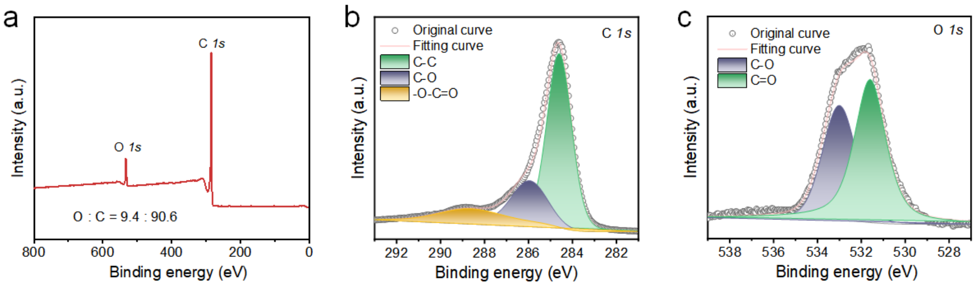

Supplementary Fig. 38 a XPS survey spectra of $MHCS_{0.5}$ after long-term electrolysis. **b**, **c** High-resolution XPS spectra of C *1s* and O *1s* of $MHCS_{0.5}$ after long-term electrolysis.

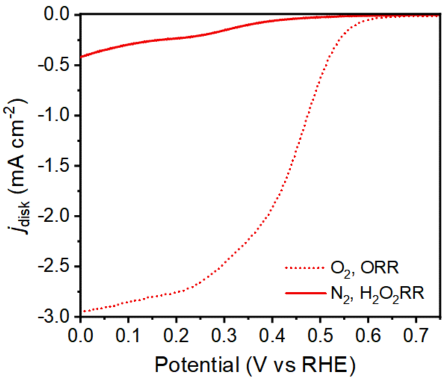

Supplementary Fig. 39 LSV curves for H_2O_2RR recorded in N_2-saturated 0.1 M PBS containing 10 mM H_2O_2.

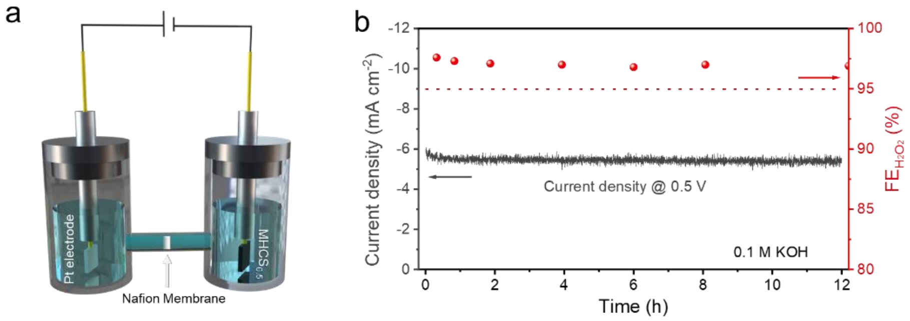

Supplementary Fig. 40 a Schematic diagram of the H-type cell setup for 2e$^-$ ORR. **b** 12-hour stability test of the bulk $MHCS_{0.5}$ at 0.5 V vs RHE on a H-type cell setup.

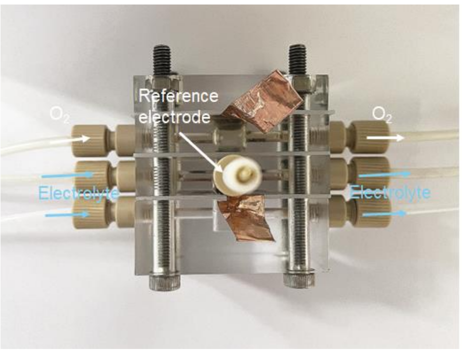

Supplementary Fig. 41 Photograph of the flow cell setup for 2e$^-$ ORR. The two-compartment flow cell, with continuous O_2 pumping and separated by a Nafion membrane, featured a cathode assembly using $MHCS_{0.5}$ catalyst ink on carbon paper, an Ag/AgCl reference electrode, commercial IrO_2 as the anode, and a 0.1 M KOH/0.1 M PBS electrolyte circulated through each compartment.

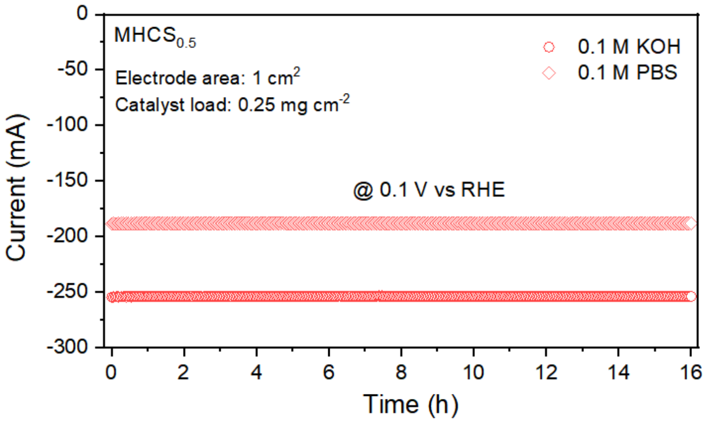

Supplementary Fig. 42 Chronoamperometry curves of current on $MHCS_{0.5}$ electrode at 0.1 V vs RHE in O_2 saturated 0.1 M KOH and 0.1 M PBS.

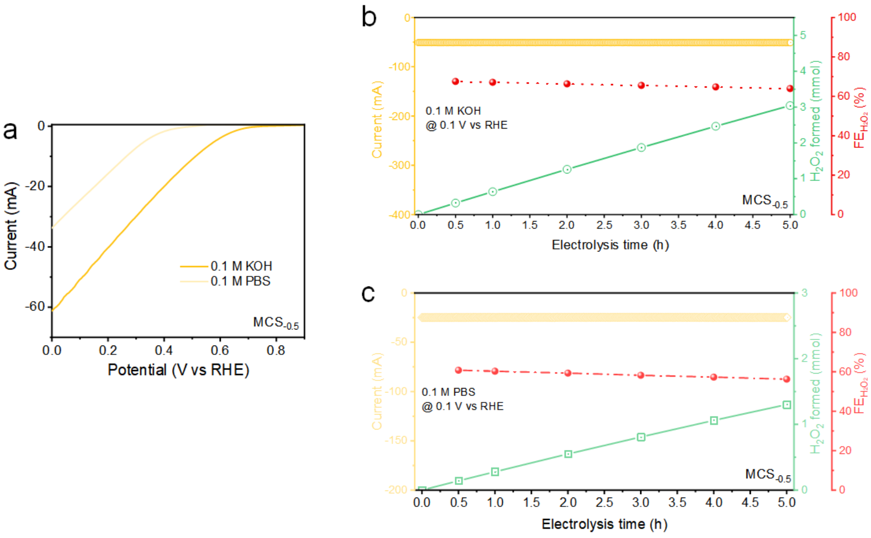

Supplementary Fig. 43 **a** Polarization curves of MCS-0.5 loaded CP electrode in flow cell. **b** Electrolysis time-dependent current, H_2O_2 production, and faradaic efficiency of MCS-0.5 under continuous O_2 purging in 0.1 M KOH. **c** Electrolysis time-dependent current, H_2O_2 production, and faradaic efficiency of MCS-0.5 under continuous O_2 purging in 0.1 M PBS.

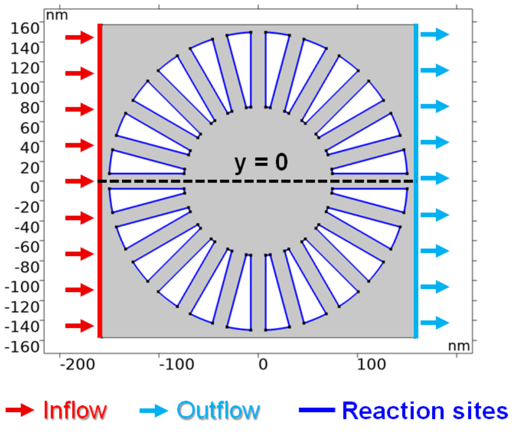

Supplementary Fig. 44 Model diagram of two-dimensional mesoporous hollow sphere.

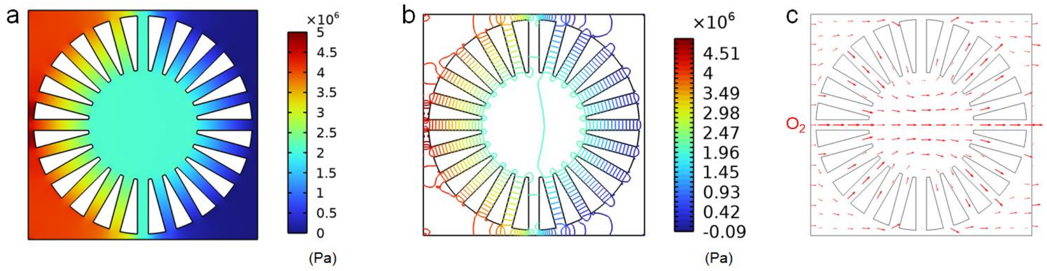

Supplementary Fig. 45 **a** Spatial distribution of pressure in a mesoporous hollow sphere model (d/r = 0, r = 150 nm, φ = 20 nm). **b** Distribution of pressure gradient across the mesoporous hollow sphere model (d/r = 0, r = 150 nm, φ = 20 nm). **c** O_2 flow path across the hollow sphere model (d/r = 0, r = 150 nm, φ = 20 nm).

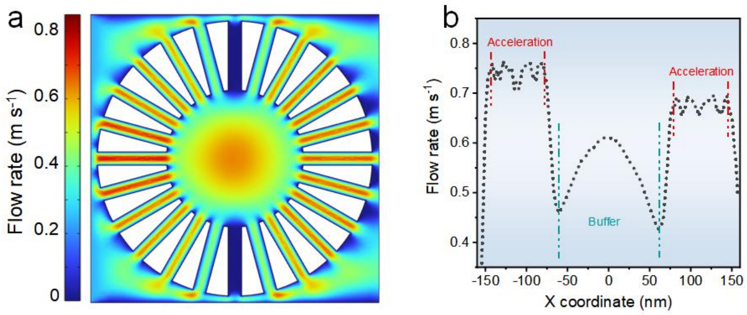

Supplementary Fig. 46 a Spatial distribution of fluid velocity in a mesoporous hollow sphere model (d/r = 0.5, r = 150 nm, φ = 20 nm). **b** Fluid velocity distribution on the truncated line (y = 0).

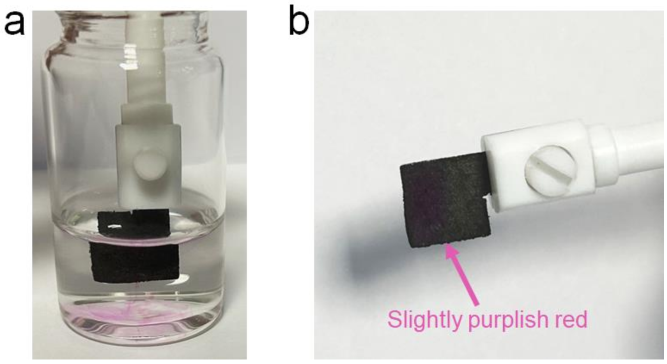

Supplementary Fig. 47 a Photograph of $MHCS_{0.5}$ electrode in phenolphthalein solution. **b** Photograph of $MHCS_{0.5}$ electrode impregnated with phenolphthalein solution.

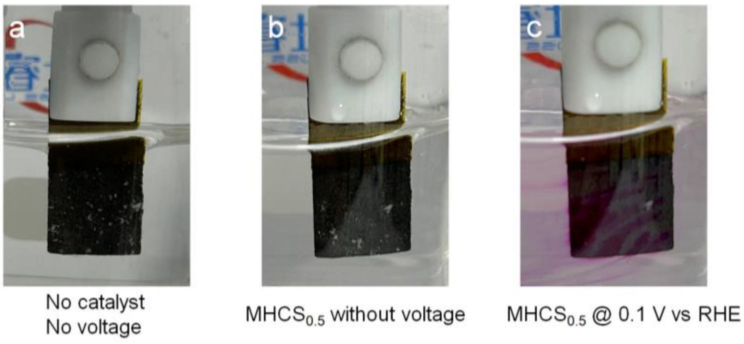

Supplementary Fig. 48 Color change of electrode surfaces in neutral electrolyte (0.1 M K_2SO_4) containing phenolphthalein: **a** carbon paper only at 0.1 V vs RHE, **b** $MHCS_{0.5}$ electrode without applied potential, **c** $MHCS_{0.5}$ Electrode at 0.1 V vs RHE.

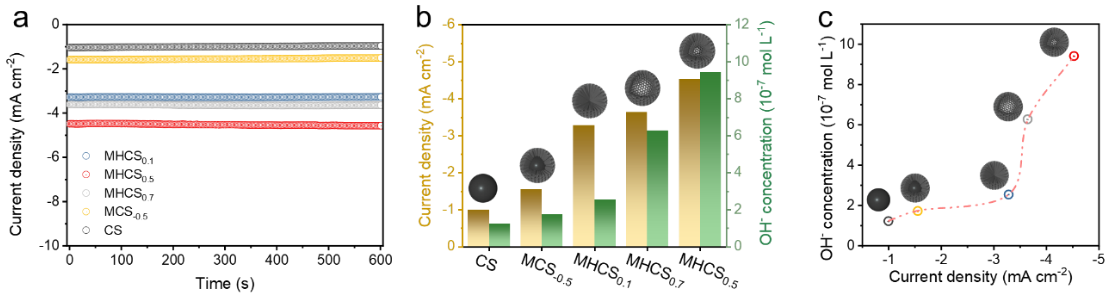

Supplementary Fig. 49 **a** Currents recorded in experiments for direct detection of the local pH changes on electrode. **b** Detected current density and OH^- concentration at the $MHCS_x$ electrode. **c** Correlation between current density and OH^- concentration detected at $MHCS_x$ electrodes.

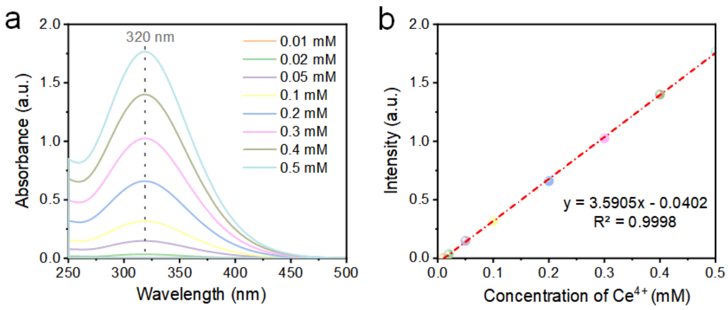

Supplementary Fig. 50 **a** UV-Vis absorption spectra of Ce^{4+} solution with various concentrations and **b** its corresponding standard curve.

Supplementary References

1. Tian Y, Li M, Wu Z, Sun Q, Yuan D, Johannessen B, *et al.* Edge-hosted atomic Co−N_4 sites on hierarchical porous carbon for highly selective two-electron oxygen reduction reaction. *Angew. Chem. Int. Ed.* **61**, e202213296 (2022).

2. Guo Y, Zhang R, Zhang S, Hong H, Zhao Y, Huang Z, *et al.* Ultrahigh oxygen-doped carbon quantum dots for highly efficient H_2O_2 production via two-electron electrochemical oxygen reduction. *Energy Environ. Sci.* **15**, 4167-4174 (2022).

3. Koh KH, Bagherzadeh Mostaghimi AH, Chang Q, Kim YJ, Siahrostami S, Han TH, *et al.* Elucidation and modulation of active sites in holey graphene electrocatalysts for H_2O_2 production. *EcoMat* **5**, e12266 (2023).

4. Wang W, Zheng Y, Hu Y, Liu Y, Chen S. Intrinsic carbon defects for the electrosynthesis of H_2O_2. *J. Phys. Chem. Lett.* **13**, 8914-8920 (2022).

5. Li L, Tang C, Zheng Y, Xia B, Zhou X, Xu H, *et al.* Tailoring selectivity of electrochemical hydrogen peroxide generation by tunable pyrrolic-nitrogen-carbon. *Adv. Energy Mater.* **10**, 2000789 (2020).

6. Chen S, Luo T, Chen K, Lin Y, Fu J, Liu K, *et al.* Chemical identification of catalytically active sites on oxygen-doped carbon nanosheet to decipher the high activity for electro-synthesis hydrogen peroxide. *Angew. Chem. Int. Ed.* **133**, 16743-16750 (2021).

7. Wu J, Hou M, Chen Z, Hao W, Pan X, Yang H, *et al.* Composition engineering of amorphous nickel boride nanoarchitectures enabling highly efficient electrosynthesis of hydrogen peroxide. *Adv. Mater.* **34**, 2202995 (2022).

8. Lim JS, Kim JH, Woo J, San Baek D, Ihm K, Shin TJ, *et al.* Designing highly active nanoporous carbon H_2O_2 production electrocatalysts through active site identification. *Chem* **7**, 3114-3130 (2021).

9. Tang C, Jiao Y, Shi B, Liu JN, Xie Z, Chen X, *et al.* Coordination tunes selectivity: two-electron oxygen reduction on high-loading molybdenum single-atom catalysts. *Angew. Chem. Int. Ed.* **132**, 9256-9261 (2020).

10. Jung E, Shin H, Lee B-H, Efremov V, Lee S, Lee HS, *et al.* Atomic-level tuning of Co–N–C catalyst for high-performance electrochemical H_2O_2 production. *Nat. Mater.* **19**, 436-442 (2020).

11. Wang Y, Shi R, Shang L, Waterhouse GI, Zhao J, Zhang Q, *et al.* High-efficiency oxygen reduction to hydrogen peroxide catalyzed by nickel single-atom catalysts with tetradentate N_2O_2 coordination in a three-phase flow cell. *Angew. Chem. Int. Ed.* **59**, 13057-13062 (2020).

12. Yang Y, Wu W, Wang Y, Liu J, Li N, Fan Y, *et al.* Enhanced electrochemical O_2-to-H_2O_2 synthesis via Cu-Pb synergistic interplay. *Small* **18**, 2106534 (2022).

13. Jiang K, Back S, Akey AJ, Xia C, Hu Y, Liang W, *et al.* Highly selective oxygen reduction to hydrogen peroxide on transition metal single atom coordination. *Nat. Commun.* **10**, 3997 (2019).

14. Yaling J, Xue Z, Yang J, Liu Q, Xian J, Zhong Y, *et al.* Tailoring the electronic structure of atomically dispersed Zn electrocatalyst by coordination environment regulation for high selectivity oxygen reduction. *Angew. Chem. Int. Ed.* **61**, e202110838 (2022).

15. Li BQ, Zhao CX, Liu JN, Zhang Q. Electrosynthesis of hydrogen peroxide synergistically catalyzed by atomic $Co–N_x–C$ sites and oxygen functional groups in noble-metal-free electrocatalysts. *Adv. Mater.* **31**, 1808173 (2019).

16. Zhang Y, Lyu Z, Chen Z, Zhu S, Shi Y, Chen R, *et al.* Maximizing the catalytic performance of $Pd@Au_xPd_{1-x}$ nanocubes in H_2O_2 production by reducing shell thickness to increase compositional stability. *Angew. Chem. Int. Ed.* **133**, 19795-19799 (2021).

17. Zhang N, Zheng F, Huang B, Ji Y, Shao Q, Li Y, *et al.* Exploring Bi_2Te_3 nanoplates as versatile catalysts for electrochemical reduction of small molecules. *Adv. Mater.* **32**, 1906477 (2020).

18. Zhang C, Liu W, Song M, Zhang J, He F, Wang J, *et al.* Pyranoid-O-dominated graphene-like nanocarbon for two-electron oxygen reduction reaction. *Appl. Catal. B* **307**, 121173 (2022).

19. Wu J, Mehmood A, Zhang G, Wu S, Ali G, Kucernak A. Highly selective O_2 reduction to H_2O_2 catalyzed by cobalt nanoparticles supported on nitrogen-doped carbon in alkaline solution. *ACS Catal.* **11**, 5035-5046 (2021).

20. Lee K, Lim J, Lee MJ, Ryu K, Lee H, Kim JY, *et al.* Structure-controlled graphene electrocatalysts for high-performance H_2O_2 production. *Energy Environ. Sci.* **15**, 2858-2866 (2022).

21. Wang Z, Li Q-K, Zhang C, Cheng Z, Chen W, McHugh EA, *et al.* Hydrogen peroxide generation with 100% faradaic efficiency on metal-free carbon black. *ACS Catal.* **11**, 2454-2459 (2021).

22. Zhang E, *et al.* Engineering the local atomic environments of indium single-atom catalysts for efficient electrochemical production of hydrogen peroxide. *Angew. Chem. Int. Ed.* **134**, e202117347 (2022).

23. Peng W, *et al.* Facilitating two-electron oxygen reduction with pyrrolic nitrogen sites for electrochemical hydrogen peroxide production. *Nat. Commun.* **14**, 4430 (2023).

24. Long Y, *et al.* Tailoring the Atomic-Local Environment of Carbon Nanotube Tips for Selective H_2O_2 Electrosynthesis at High Current Densities. *Adv. Mater.* 2303905 (2023).

25. Li Y, *et al.* Single-atom Iron Catalyst with Biomimetic Active Center to Accelerate Proton Spillover for Medical-level Electrosynthesis of H_2O_2 Disinfectant. *Angew. Chem. Int. Ed.* e202306491 (2023).

26. Fan M, *et al.* N-B-OH Site-Activated Graphene Quantum Dots for Boosting Electrochemical Hydrogen Peroxide Production. *Adv. Mater.* 2209086 (2023).

27. Xiang F, *et al.* Enhanced Selectivity in the Electroproduction of H_2O_2 via F/S Dual-Doping in Metal-Free Nanofibers. *Adv. Mater.* 2208533 (2022).

28. Wu J, *et al.* The electron transport regulation in carbon dots/In_2O_3 electrocatalyst enable 100% selectivity for oxygen reduction to hydrogen peroxide. *Adv. Funct. Mater.* **32**, 2203647 (2022).

29. Chen Z, *et al.* Entropy enhanced perovskite oxide ceramic for efficient electrochemical reduction of oxygen to hydrogen peroxide. *Angew. Chem. Int. Ed.* **134**, e202200086 (2022).

30. Qi D, *et al.* Cyclodextrin-supported $Co(OH)_2$ Clusters as Electrocatalysts for Efficient and Selective H_2O_2 Synthesis. *Angew. Chem. Int. Ed.* **135**, e202307355 (2023).

31. Sun L, Jin X, Su T, Fisher AC, Wang X. Conjugated Nickel Phthalocyanine Derivatives for Heterogeneous Electrocatalytic H_2O_2 Synthesis. *Adv. Mater.* 2306336 (2023).

32. Xia Y, *et al.* Highly active and selective oxygen reduction to H_2O_2 on boron-doped carbon for high production rates. *Nat. Commun.* **12**, 1-12 (2021).

33. Lu ZY, *et al.* High-efficiency oxygen reduction to hydrogen peroxide catalysed by oxidized carbon materials. *Nat. Catal.* **1**, 156-162 (2018).

34. Gao M, Wang Z-Y, Yuan Y-R, Li W-W, Liu H-Q, Huang T-Y. Ball-milled biochar for efficient neutral electrosynthesis of hydrogen peroxide. *Chem. Eng. J.* **434**, 134788 (2022).

35. Zhang Y, *et al.* Metastable Hexagonal Phase SnO_2 Nanoribbons with Active Edge Sites for Efficient Hydrogen Peroxide Electrosynthesis in Neutral Media. *Angew. Chem. Int. Ed.* e202218924 (2023).

36. Lee B-H, *et al.* Supramolecular tuning of supported metal phthalocyanine catalysts for hydrogen peroxide electrosynthesis. *Nat. Catal.* **6**, 234-243 (2023).

37. Yu Z, *et al.* Low-Coordinated Pd Site within Amorphous Palladium Selenide for Active, Selective, and Stable H_2O_2 Electrosynthesis. *Adv. Mater.* **35**, 2208101 (2023).

38. Zhang S, *et al.* Tafel Analysis Guided Optimization of ZnNP-OC Catalysts for the Selective 2-Electron Oxygen Reduction Reaction in Neutral Media. *J. Phys. Chem. Lett.* **13**, 3409-3416

(2022).

39. Ding S, *et al.* An abnormal size effect enables ampere-level O_2 electroreduction to hydrogen peroxide in neutral electrolytes. *Energy Environ. Sci.* **16**, 3363-3372 (2023).

40. Zhou Y, *et al.* The operation active sites of O_2 reduction to H_2O_2 over ZnO. *Energy Environ. Sci.* **16**, 3526-3533 (2023).

41. Zhang C, *et al.* Crystal engineering enables cobalt-based metal–organic frameworks as high-performance electrocatalysts for H_2O_2 production. *J. Am. Chem. Soc.* **145**, 7791-7799 (2023).

42. Tian Z, *et al.* Constructing Interfacial Boron-Nitrogen Moieties in Turbostratic Carbon for Electrochemical Hydrogen Peroxide Production. *Angew. Chem. Int. Ed.* **134**, e202206915 (2022).

43. Sun Y, *et al.* Activity–selectivity trends in the electrochemical production of hydrogen peroxide over single-site metal–nitrogen–carbon catalysts. *J. Am. Chem. Soc.* **141**, 12372-12381 (2019).

44. Zhang H-X, *et al.* Electrocatalyst derived from fungal hyphae and its excellent activity for electrochemical production of hydrogen peroxide. *Electrochim. Acta* **308**, 74-82 (2019).

45. Fu H, *et al.* Lattice Strained B-Doped Ni Nanoparticles for Efficient Electrochemical H_2O_2 Synthesis. *Small* **18**, 2203510 (2022).

46. Yan M, *et al.* Sb_2S_3-templated synthesis of sulfur-doped Sb-NC with hierarchical architecture and high metal loading for H_2O_2 electrosynthesis. *Nat. Commun.* **14**, 368 (2023).

47. Zhao Q, *et al.* Approaching a high-rate and sustainable production of hydrogen peroxide: oxygen reduction on Co–N–C single-atom electrocatalysts in simulated seawater. *Energy Environ Sci.* **14**, 5444-5456 (2021).

48. Yang Z, Gao Y, Zuo L, Long C, Yang C, Zhang X. Tailoring Heteroatoms in Conjugated Microporous Polymers for Boosting Oxygen Electrochemical Reduction to Hydrogen Peroxide. *ACS Catal.* **13**, 4790-4798 (2023).

49. Zhou Y, *et al.* Efficient synthesis of H_2O_2 via oxygen reduction over PANI driven by kinetics regulation of carbon dots. *Appl. Catal. B* **322**, 122105 (2023).

50. De Boer J, Lippens B, Linsen B, Broekhoff J, Van den Heuvel A, Osinga TJ. The t-curve of multimolecular N_2-adsorption. *J. Colloid Interf. Sci*. **21**, 405-414 (1966).
