# Supplementary material for: Mesoporous carbon spheres with programmable interiors as efficient nanoreactors for H2O2 electrosynthesis
Source: Nat Commun. 2024 Feb 1;15:983. doi: 10.1038/s41467-024-45243-w (PMC10834542; doi:10.1038/s41467-024-45243-w)
Supplement: Supplementary file 3 — Description of Additional Supplementary Files [file 41467_2024_45243_MOESM3_ESM.pdf]

DESCRIPTION OF ADDITIONAL SUPPLEMENTARY FILES DOCUMENT

Supplementary Movie 1 - Direct in-situ observation of localized pH elevation. Time-dependent color evolution of MHCS0.5 electrode surface (at 0.1 V vs RHE) in neutral electrolyte (0.1 M K2SO4) containing phenolphthalein.
